# Recent Advances in Sequentially Pd-Catalyzed One-Pot Syntheses of Heterocycles

**DOI:** 10.3390/molecules29225265

**Published:** 2024-11-07

**Authors:** Maryna M. Kornet, Thomas J. J. Müller

**Affiliations:** 1 Institut für Organische Chemie und Makromolekulare Chemie, Mathematisch-Naturwissenschaftliche Fakultät, Heinrich-Heine-Universität Düsseldorf, Universitätsstrasse 1, D-40225 Düsseldorf, Germany; 2 Laboratory for Biotechnology of Physiologically Active Substances, Faculty of Biology, Zaporizhzhia National University, 66 Universytetska Str., 69600 Zaporizhzhia, Ukraine

**Keywords:** catalysis, heterocycles, one pot, palladium, sequential

## Abstract

Sequential Pd-catalyzed one-pot synthetic methodologies have emerged as a powerful and versatile approach in organic synthesis, enabling the construction of complex heterocyclic architectures with high efficiency, selectivity, and atom economy. This review discusses key advancements in multistep, sequentially Pd-catalyzed one-pot processes for accessing heterocyclic derivatives, focusing on classic reactions like Suzuki–Miyaura, Sonogashira, Heck, and hydroamination and extending to specialized techniques such as directed C-H activation. The concatenation of these steps has advanced the scope of one-pot strategies. A section is dedicated to exploring the cooperative use of palladium with other metals, particularly copper, ruthenium, and gold, which has broadened the range of accessible heterocyclic derivatives. Highlighted applications include the synthesis of biologically and pharmaceutically relevant compounds, such as tris(hetero)aryl systems, spiro-oxindoles, and indole derivatives. These one-pot strategies not only streamline synthesis but also align with green chemistry principles by minimizing purification steps and reducing waste and energy consumption. The review also addresses current challenges and limitations in these methodologies, offering insights into ongoing efforts to optimize reaction conditions and expand the applicability of sequential Pd-catalyzed processes.

## 1. Introduction

Palladium catalysis has long served as a fundamentally important cornerstone of synthetic organic chemistry, defining its standard practices for more than half a century. Renowned for its capacity to form multiple carbon–carbon (C–C) and carbon–heteroatom (C–X) bonds in a single operation, these reactions encompass a diverse array of organic transformations, particularly cross-couplings, aminations, and conversions involving directed C-H activation [1,2]. These processes provide access to versatile complex molecular architectures, often under mild conditions and with high yields [3,4]. The broad application scope and variety of scenarios underpin the strength of modern total synthesis and play a crucial role in applied organic chemistry, particularly in the production of high-value-added pharmaceuticals and functional materials [5,6,7,8]. Palladium-catalyzed synthetic methodologies align with the principles of green chemistry, as they offer opportunities for enhanced recoverability and address the need to minimize hazardous chemicals and by-products, reduce the number of steps, improve atom economy [9], and optimize energy expenditure, all of which contribute to environmentally sustainable efficiency [10].

One of the foundational strategies for improving efficiency in synthesis is to combine multiple reaction steps in sequences within a one-pot process, thereby eliminating the need for multiple purifications and significantly shortening the overall synthetic time while potentially increasing yields [11]. Specifically, the concept of sequential catalysis suggests that a single catalyst, often a metal complex, facilitates not just one but several consecutive transformations [12]. The one-pot approach implies that all transformations within a sequential catalytic process occur in the same reaction vessel, eliminating the need for intermediate workups, isolations, or solvent changes. This in sensu stricto definition of one-pot methodologies aligns with concepts such as domino and multicomponent reactions, as described by Tietze [13,14]. Accordingly, one-pot processes may proceed either in a domino fashion (with all reactants, catalysts, and additives present from the beginning) or consecutively (where reactants and additives are added step by step). It is worth noting that domino cascades, in a narrow sense, might also be understood as a sequence that takes place under identical conditions throughout the complete sequence [15,16,17]. Regarding palladium catalysis, its unparalleled versatility renders it particularly promising for multiply catalyzed one-pot sequences, referred to here as sequentially Pd-catalyzed processes. Over the past few decades, significant progress has been made in expanding the scope of Pd-catalyzed sequences. More versatile substrates and functional groups have been tested to facilitate the synthesis of compounds with potential biological activity or functional material applications driven by structural diversity-oriented activities [18,19,20]. Recent reviews on processes with Pd catalysis involving more than one step have been reported [7,21], besides our overviews in 2007 and 2015 [22,23].

In this review, we provide a comprehensive overview of the literature published since 2015 on the synthesis of complex heterocyclic compounds, emphasizing considerations of green chemistry and general efficiency, as well as some selected cases from earlier studies not previously reviewed. The core part of the review focuses on processes with more than one sub-step involved in catalysis using palladium (including cases where synergistic additions of other metal catalysts, e.g., copper, are involved). The second part of the review examines combinations with other metal catalysts where only one step is used to perform catalysis using palladium.

## 2. Sequences with Two or More Steps Catalyzed by Palladium

The organization of sub-sections follows a logical progression, moving from more common reactions to specialized ones, with the C-H activation positioned as the most interesting standard case from the perspective of general organic synthesis. Given the prevalence of the Suzuki–Miyaura reaction in the reviewed literature, it was practical to initiate the discussion with sequences centered around this reaction. The frequent Suzuki–Masuda combination was introduced under the umbrella of Suzuki–Miyaura reactions. Classification of a given sequence under a specific sub-section generally reflects the order of the reactions within the sequence, with each subsequent reaction establishing a natural hierarchy. However, this ordering was not applied rigidly; for certain significant sequences, placements were adjusted to enhance clarity and provide a more intuitive understanding for the reader. Additionally, certain relay, cascade, and domino reactions—processes where sub-steps proceed without any intermediate change in conditions—are also included in the discussion.

### 2.1. Sequentially Pd-Catalyzed Processes Based upon Suzuki–Miyaura Cross-Coupling

The palladium-catalyzed Suzuki–Miyaura cross-coupling (hereafter referred to as the Suzuki reaction/coupling) remains one of the most reliable methods for creating carbon–carbon bonds [24]. The reaction between organohalides and organoboronic acids accommodates not only the commonly used aryl derivatives but also alkyl, alkenyl, or alkynyl groups as at least one of the coupling partners. Since its inception in 1979, the scope of the Suzuki reaction has continuously expanded, and today, it is considered a primary contender for the title of one of the most essential transformations in organic synthesis. The well-investigated, already classical mechanism of the Suzuki reaction involves the oxidative addition of a Pd^0^ species to a C-Hal bond, and subsequent ligand exchange to form an organopalladium intermediate, transmetalation, and reductive elimination is amply covered by excellent specialized reviews together with the recent advances in the field [2,25]. This review focuses on the integration of the Suzuki reaction into one-pot reaction sequences, which may further enhance its importance due to the robustness and functional group compatibility of the employed precursor.

**Scheme 1:**
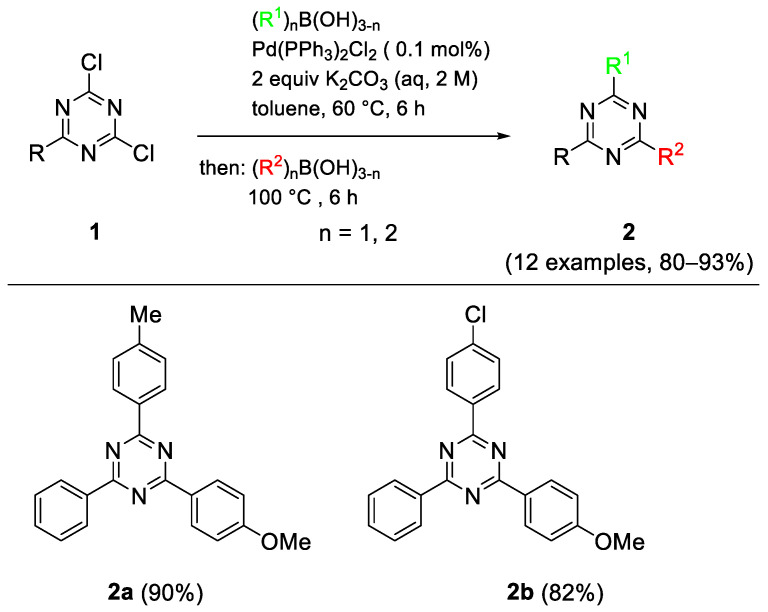
One-pot synthesis of unsymmetrically trisubstituted aryl *s*-triazines **2** via sequential Suzuki coupling [26].

The article by C. Wang and colleagues presents a practical and highly selective method for synthesizing unsymmetrical aryl-s-triazines **2** via a sequential Suzuki coupling [26]. The substitution of the first chloro substituent among the two equivalent chloro groups in cyanuric chloride **1** (2,4,6-trichlorotriazine) reduces the reactivity of the remaining one, thereby avoiding over-substitution. The coupling is catalyzed by 0.1–0.5 mol% Pd(PPh_3_)_2_Cl_2_, with the first step conducted at 60 °C and the second at 100 °C (Scheme 1). The authors demonstrate that optimizing reaction conditions such as selecting toluene as the solvent and maintaining anhydrous conditions, allows for controlled mono-, di-, and trisubstitution. The approach yields trisubstituted aryl-*s*-triazines in a one-pot procedure with high efficiency. The study also underscores the role of electronic effects: electron-withdrawing groups on aryl boronic acids can hinder the coupling process, whereas steric hindrance from electron-donating *ortho*-substituents can be effectively managed.

While this example is one of the simplest, it illustrates an efficient high-yield protocol for synthesizing trisubstituted *s*-triazines with completely different aryl substituents using a symmetric dihalo-precursor.

**Scheme 2:**
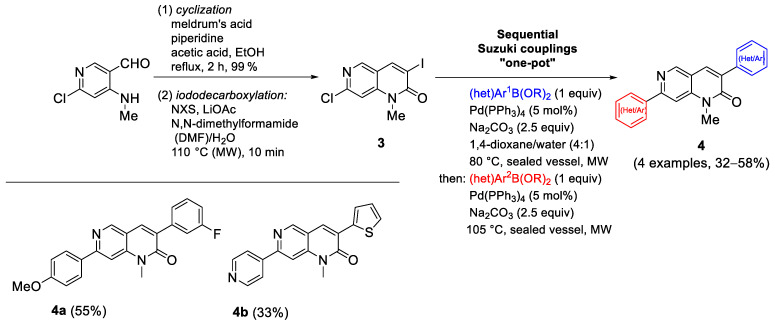
One-pot sequential Suzuki–Miyaura couplings of 7-chloro-3-iodo-1,6-naphthyridin-2(1*H*)-ones **3** [27].

In contrast to the previous case, which used two equivalent halogens, Montoir et al. employed an asymmetric chloro–iodo precursor **3** for the realization of a rapid and efficient strategy of differential functionalization of 1,6-naphthyridin-2(1*H*)-ones **4** through sequential, site-selective one-pot Suzuki–Miyaura cross-coupling reactions [27]. The distinguishing aspect of this work is the concise synthesis of the 1,6-naphthyridin-2(1*H*)-one precursor via the iododecarboxylation of 7-chloro-3-iodo-1-methyl-1,6-naphthyridin-2(1*H*)-one **3** (Scheme 2). This key intermediate serves as a versatile reagent for microwave-assisted Pd-catalyzed cross-coupling reactions, significantly reducing reaction times and improving yields. The methodology facilitates the synthesis of highly functionalized 3,7-diaryl-substituted naphthyridones, with potential pharmaceutical applications. The authors suggest that this approach can be extended to other metal-catalyzed reactions for direct functionalization at both C-3 and C-7 positions of this readily accessible scaffold.

Although the concept is straightforward, maximally differentiating the halo substituents, the efficient and succinct realization of the sequence is what distinguishes this work.

**Scheme 3:**
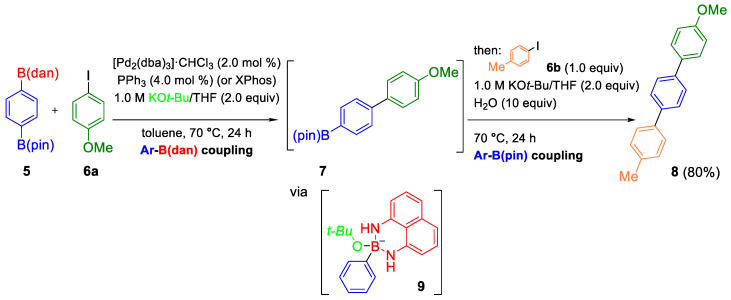
One-pot iterative Suzuki coupling of dan- and pin-derivatized difunctional arylboronic acids **5** with two different aryl iodides (**6a** and **6b**) in a sequence [28].

Contrary to the previous situations, when the core precursor was a bis-halogenide, Mutoh et al. utilized an asymmetric bis-arylboronic acid, following the same general idea of a Suzuki–Suzuki sequence [28]. The 1,8-diaminonaphthalene (dan)-protected arylboronic acid **7**, in the presence of *t*-BuOK as a base, enables effective transmetalation and, eventually, cross-coupling without the need for deprotection (Scheme 3). The standard catalytic cycle involves an arylpalladium (II) complex, which subsequently exchanges the halide for *tert*-butylate, and the resulting butoxide complex **9** participates in transmetalation with the boronate. The authors note that potassium ions may enhance the process through weak cation–π interaction. The reductive elimination of the diaryl palladium (II) intermediate, regenerating the palladium(0) catalyst, yields the biaryl product **7** with a good yield. This protocol was extended to a one-pot sequential Suzuki–Miyaura cross-coupling of 4-[(pin)B]C_6_H_4_–B(dan) **5**, selectively coupling the less reactive aryl-B(dan) moiety first, followed by a second cross-coupling step. This strategy exemplifies the method’s versatility and step economy, streamlining the synthesis of complex biaryl structures **8** and overcoming traditional limitations associated with boronic acid reactivity.

**Scheme 4:**
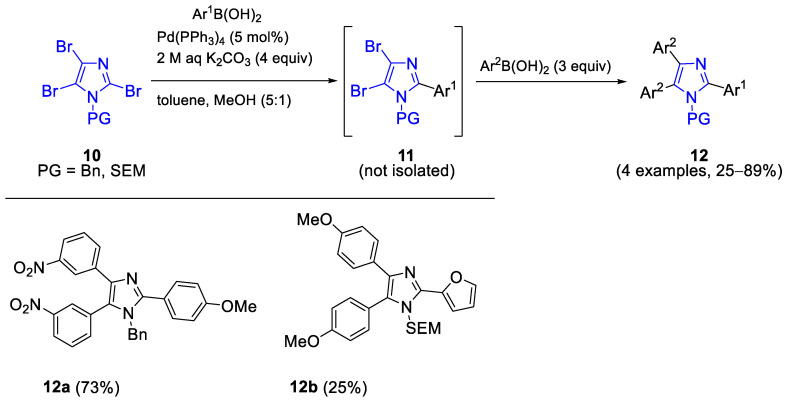
One-pot synthesis of triarylated imidazoles via Suzuki–Miyaura cross-coupling [29].

A further increase in the number of substitution positions to three by a two sub-step sequence of Suzuki couplings was demonstrated by Recnik et al. [29]. The process involves stepwise arylation of 1-protected 2,4,5-tribromoimidazole **10**, starting with selective arylation at the second position **11**, followed by additional arylations at positions 4 and 5, achieving high regioselectivity and overall efficiency (Scheme 4). The strategy successfully provides access to a variety of 2,4,5-triarylated imidazoles **12**, including neurodazine—a compound known for its promising neuronal cell-differentiating properties and for its potential as a therapeutic agent. The reported approach is particularly valuable in the medicinal chemistry context for generating complex drug-candidate molecules with high regioselectivity.

**Scheme 5:**
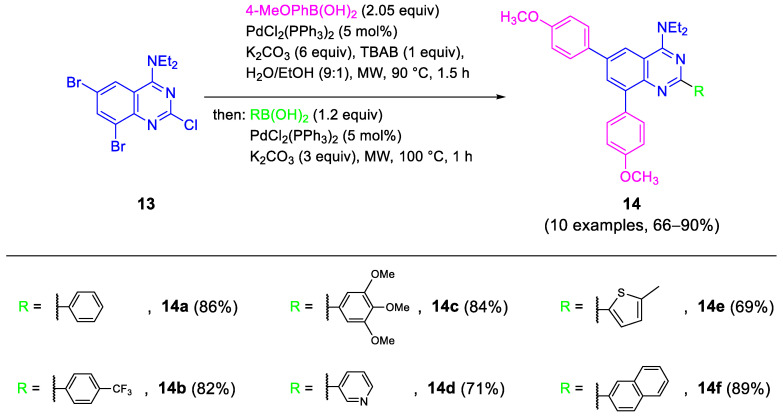
One-pot chemoselective synthesis of 2,6,8-trisubstituted 4-aminoquinazoline derivatives **14** via threefold Suzuki coupling [30].

Using different halogens in a situation similar to the previous case allows for even more precise control over regioselectivity, as demonstrated in the efficient strategy leading to 2,6,8-trisubstituted 4-amino quinazolines **14** [30]. The synthesis begins with the selective amination of 6,8-dibromo-2,4-dichloroquinazoline **13** at C-4 using diethylamine or 3-chloroaniline as a separate step. It is followed by a two sub-step threefold Suzuki coupling sequence, using a couple of different boronic acids, such as (4-methoxyphenyl)boronic acid, and phenylboronic acid, to introduce substituents at the C-2, C-6, and C-8 positions (Scheme 5). The process, conducted in an aqueous medium with Pd(PPh_3_)_2_Cl_2_ as a catalyst, K_2_CO_3_ as a base, and TBAB as a phase-transfer catalyst under microwave irradiation at 90–100 °C, ensures high chemoselectivity and efficient conversion. This method yields disubstituted derivatives at the C-6 and C-8 positions and completes the trisubstituted at the C-2 position with up to 86% yield, all while maintaining environmental friendliness and broad tolerance for various substituents.

**Scheme 6:**
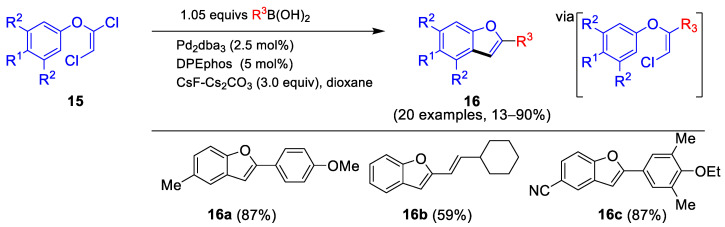
One-pot preparation of 2-substituted benzo[*b*]furans **16** from symmetrical phenols **1** [31].

The previously discussed examples exclusively employed Suzuki sequences. More advanced cases could be carried out when the Suzuki coupling is only one preparative step. Thus, Geary et al. developed a one-pot sequential Suzuki cross-coupling followed by a direct arylation, leading to intermolecular hetero-cyclization for the efficient synthesis of 2-substituted benzo[*b*]furans **16** from simple phenols, boronic acids, and trichloroethylene [31]. This process involves two synthetic steps with the key step being a sequential Pd-catalyzed reaction that tolerates a wide range of functional groups without requiring reactivity-enhancing “activating” functionalities. The Suzuki cross-coupling occurs at the C^1^–Cl position of dichlorovinylaryl ethers **15**, followed by heterocyclization to form benzo[*b*]furans **16** (Scheme 6). Kinetic isotope effect studies and regioselectivity investigations suggest that the direct arylation does not involve an electrophilic palladation mechanism. Instead, the mechanism likely involves an intermediary mechanism between C–H bond metathesis and an assisted palladation or concerted metalation–deprotonation pathway. This versatile and modular approach provides rapid access to diverse benzofuran derivatives, highlighting its potential in drug development.

**Scheme 7:**
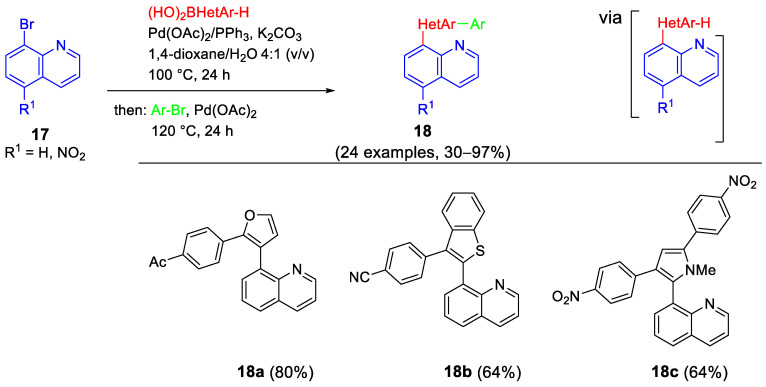
One-pot synthesis of the 8-heteroarylquinolines **18** via Pd-catalyzed sequential Suzuki–Miyaura cross-coupling/C–H arylation reaction [32].

Brodnik et al. reported an efficient one-pot synthesis of 8-heteroaryl substituted quinolines **18** through a sequential Pd-catalyzed Suzuki–Miyaura coupling followed by directed C–H functionalization [32]. This approach introduces various pyrrolyl, furanyl, thiophenyl, and benzo analogs at the 8-position of the quinoline nucleus. The method involves an initial Suzuki–Miyaura coupling with an electron-rich five-membered heterocycle (including benzannulated variants), followed by direct C–H functionalization, allowing for the efficient preparation of various 8-heteroaryl substituted quinolines, where the heteroaryl group is 2- and/or 3/4-arylsubstituted (benzo)furan or (benzo)thiophene, as well as *N*-methylpyrrole (Scheme 7). Optimized reaction conditions using Pd(OAc)_2_ and SPhos as a ligand gave high yields for furan, thiophene, and 1-methyl-1*H*-pyrrole derivatives, while benzo[*b*]furan and benzo[*b*]thiophene required alternative conditions to minimize dimerization, such as using a large excess of heteroarene or specific solvent mixtures. Due to limitations in the directed arylation of unsubstituted five-membered heteroarenes, alternative catalytic systems were explored, with the model reaction between 8-bromoquinoline **17** and (furan-3-yl)boronic acid yielding the best results using Pd(OAc)_2_/PPh_3_ and K_2_CO_3_ as a base in a 1,4-dioxane/water mixture at 100 °C. Despite testing microwave (MW) assistance, no significant improvements were observed over conventional heating. A notable application of this methodology was a three-step one-pot transformation, which adds a hydrogenation step, yielding the final product 8-[3-(4-aminophenyl)benzofuran-2-yl]quinolin-5-amine with a good overall yield. These sequential Pd-catalyzed reactions offer efficient access to hetero(aryl) quinolines with potential applications as cathepsin inhibitors and noteworthy photophysical and electrochemical properties.

**Scheme 8:**
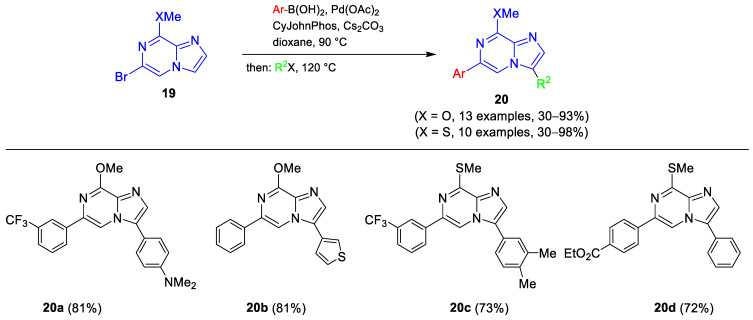
Palladium-catalyzed one-pot sequential Suzuki cross-coupling; direct C–H functionalization of imidazo[1,2-*a*]pyrazines **19** [33].

The Suzuki coupling serves as a convenient entry to more specialized transformations. Gembus et al. developed an efficient and selective methodology for the C3/C6 functionalization of imidazo[1,2-*a*]pyrazines **19** via a palladium-catalyzed Suzuki–Miyaura cross-coupling, followed by direct C–H arylation, vinylation, or benzylation [33]. This approach is remarkable for its ability to maintain catalytic activity even in the presence of methyl thioethers—traditionally inhibitory groups, including one at the C8 position. Under the proposed conditions, these groups do not interfere, enabling a third cross-coupling step to generate 3,6,8-trisubstituted imidazo[1,2-*a*]pyrazines **20** (Scheme 8).

However, it is important to note that this third step does not occur in a one-pot format but rather as a separate reaction following the initial functionalizations. This strategy enables the rapid assembly of a diverse array of mono-, di-, and trifunctionalized heterocycles, which are of considerable interest for pharmaceutical applications. Additionally, this work highlights one of the rare successful examples of directed C–H arylation with heteroaryl halides containing sulfide groups, broadening the scope of Pd-catalyzed reactions in heterocyclic chemistry.

**Scheme 9:**
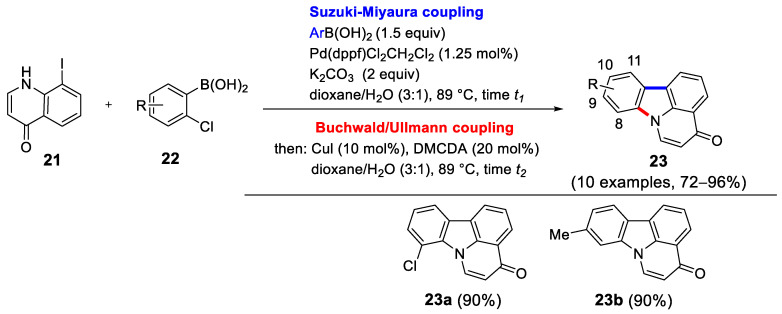
One-pot, two-step synthesis of 3-deazacanthin-4-ones **23** [34].

The Suzuki reaction can be productively paired with aminations, and the following examples demonstrate the utility of this combination: Broumidis et al. reported the synthesis of 3-deazacanthin-4-one **23** and its analogs via an efficient one-pot process involving sequential Pd-catalyzed Suzuki and intermolecular Buchwald–Hartwig/Ullmann reactions [34]. The method utilized accessible 8-iodoquinolones **21** and 2-chloroarylboronic acids **22** to construct the central pyrrolic ring, achieving high yields (Scheme 9). The intermolecular amination sub-step is synthetically notable as CuI is introduced as a co-catalyst.

The mild reaction conditions indicate a primarily Pd-catalyzed (i.e., Buchwald–Hartwig) mechanism rather than a typical Ullmann amination. Thus, the copper catalyst’s presumed auxiliary role led to the formal assignment of this sequence as a Pd-only catalyzed case. This approach proved effective for synthesizing 8-, 9-, and 10-substituted 3-deazacanthin-4-ones **23**, although steric hindrance posed challenges for certain substituents. The authors had previously worked on the synthesis of canthin-6 ones using a similar methodology, which involved unorthodox central pyrrolic ring formation [35]. The synthesized compounds exhibited promising biological activities, including the inhibition of cGMP phosphodiesterase and SR protein-phosphorylating CLK kinases, highlighting their potential therapeutic applications.

**Scheme 10:**
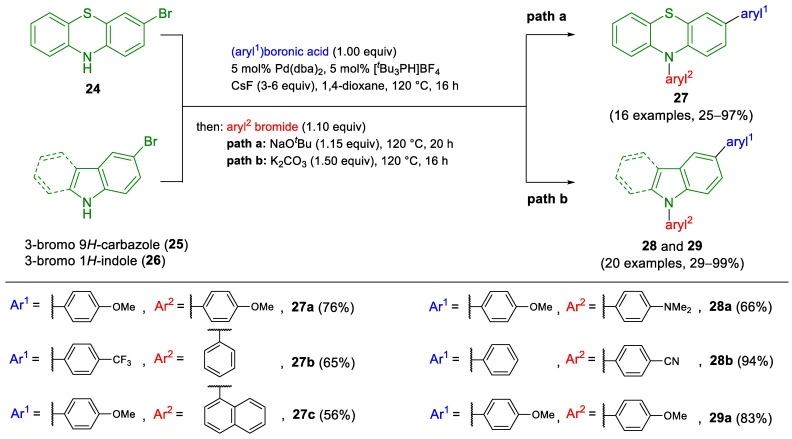
Sequential Pd-catalyzed arylation–amination three-component synthesis of 3,10-diaryl-10*H*-phenothiazines **27**, 3,9-diaryl-9*H*-carbazoles **28**, and 1,5-diaryl-1*H*-indoles **29** [36,38].

The Müller group developed a one-pot, three-component synthetic methodology combining Suzuki coupling with Buchwald–Hartwig amination, facilitating the efficient formation of C,N-diarylated heterocycles such as phenothiazines **27**, carbazoles **28**, and indoles **29** [36]. A noteworthy feature of this sequence is that the homocoupling of 3-bromo-10*H*-phenothiazine was prevented by adding cesium fluoride, which made the Suzuki coupling selective (Scheme 10). Maintaining anhydrous conditions during Suzuki coupling was crucial, as even trace amounts of water significantly reduced the efficiency of the subsequent Buchwald–Hartwig amination. The optimized conditions, involving Pd(dba)_2_ and [*t*Bu_3_PH]BF_4_ as catalysts at 120 °C, yielded highly functionalized heterocycles in moderate to very excellent yields, demonstrating the efficiency and versatility of the sequential one-pot approach.

Building on these results, the concept was extended to encompass a wider range of 3,10-diaryl phenothiazines [37]. The authors refined the synthetic approach and conducted a detailed analysis of the electrochemical and photophysical properties of these derivatives, offering deeper insights into the structure–property relationships. These results enable the rational design of phenothiazines with tailored oxidation potentials and photophysical characteristics.

**Scheme 11:**
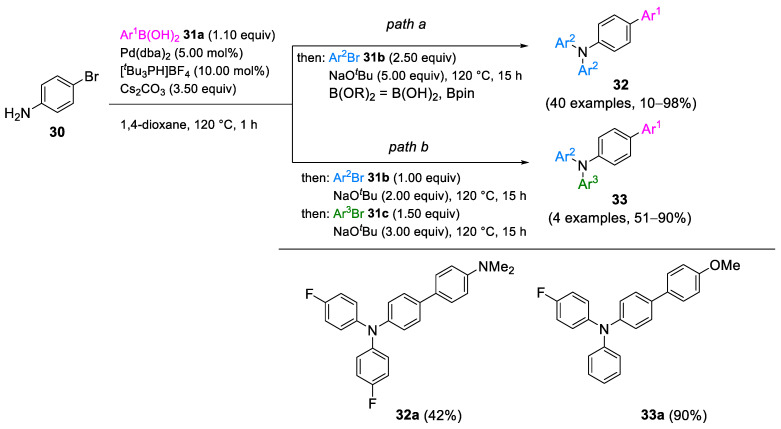
Consecutive pseudo-four-component synthesis of *sym-p*-bTAAs **32** (path a) and consecutive four-component synthesis of *unsym-p*-bTAAs **33** (path b) via Suzuki coupling and Buchwald–Hartwig amination sequences [38].

Extending this approach to other molecular frameworks, Kohlbecher et al. described a concise method for synthesizing *para*-biaryl-substituted triarylamines (*p*-bTAAs) through a sequentially Pd-catalyzed Suzuki coupling followed by twofold Buchwald–Hartwig aminations [38]. The reaction sequence begins with coupling 4-bromoaniline **30** and aryl boronic acids **31** under Pd(dba)_2_ catalysis, followed by Buchwald–Hartwig aminations using NaO*t*Bu as a base (Scheme 11). This method efficiently produces *p*-bTAAs (**32** and **33**) with a variety of electronic properties, demonstrating their significant potential for use in organic light-emitting diodes (OLEDs) and other electronic materials.

The sequences discussed above primarily begin with Suzuki coupling. Meanwhile, one of the simplest related Pd-catalyzed sequences is the Masuda borylation–Suzuki coupling (MBSC), which involves an initial borylation of an aryl halide to produce a boronic acid derivative (i.e., a “pre-Suzuki” step) followed by a Suzuki coupling between an aryl or vinyl halide [2,39]. A related but more specific variation is the Masuda borylation–Suzuki arylation (MBSA), which specifically focuses on aryl–aryl bond formation. The rest of this review will be devoted to sequences where MBSA serves as the principal conversion.

**Scheme 12:**
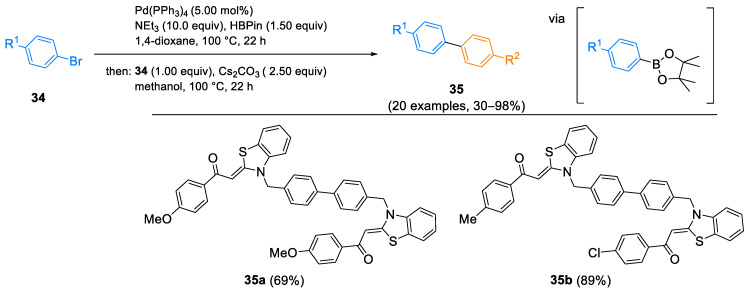
One-pot MBSA (Masuda borylation–Suzuki arylation) synthesis of biphenylene bridged bisaroyl-*S*,*N*-ketene acetal **35** [40].

Biesen et al. demonstrated a straightforward yet efficient strategy for synthesizing symmetric or near-symmetric biaryls, namely biphenylene-bridged bisaroyl-*S*,*N*-ketene acetals **35**, by preparing 20 compounds aimed at tuning their solid-state emission and aggregation-induced enhanced emission (AIEE) characteristics [40]. The MBSA coupling sequence enables direct access to these acetals in 30–98% yields from aroyl-*S*,*N*-ketene acetal monomers, eliminating the need for isolating intermediary borylated compounds (Scheme 12). This methodology accommodates a variety of substituents, including electron-donating groups like dimethylamino and methoxy, and electron-withdrawing groups such as halides and cyano groups, leading to higher yields with electron-donating substituents. Additionally, some of the synthesized compounds have been proposed as effective fluorometric probes for detecting water content in alcoholic beverages, demonstrating their potential in analyte screening based on distinct AIEE properties.

**Scheme 13:**
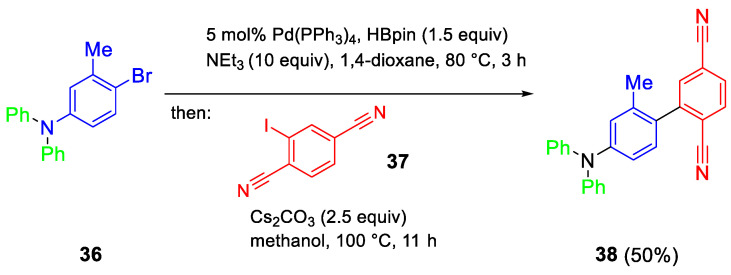
Synthesis of a donor–acceptor biaryl TADF **38** dye by sequentially Pd-catalyzed Masuda borylation–Suzuki arylation (MBSA) sequence [42].

Another example of biaryl synthesis using the Masuda–Suzuki sequence was provided by Sommer et al., who obtained donor–acceptor biaryl systems targeting thermally activated delayed fluorescence (TADF) (Scheme 13) [41]. Pd-catalyzed Masuda borylation with pinacolyl borane **36**, followed by Suzuki arylation with 2-iodoterephthalonitrile **37**, produced a twisted biaryl chromophore **38** in 50% yield in a one-pot process. This chromophore exhibits pronounced TADF with high photoluminescence quantum yield, indicating its potential for OLED technology.

**Scheme 14:**
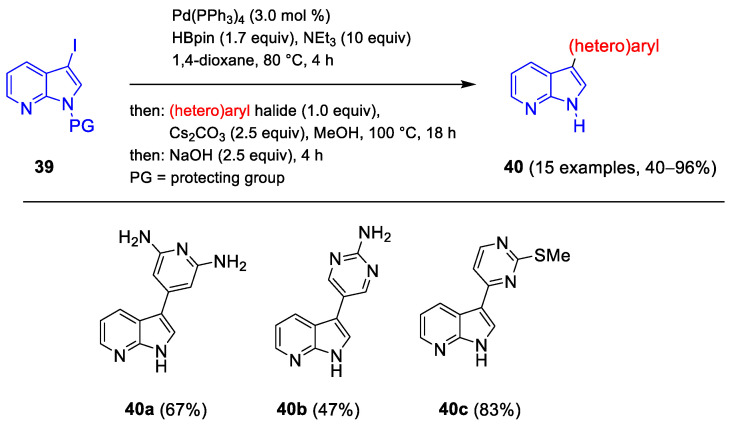
Masuda borylation–Suzuki (hetero)arylation (MBSA) sequence; one-pot synthesis of meriolin derivatives **40** [42].

Drießen et al. reported the MBSA synthesis of 3-(hetero)aryl substituted 7-azaindoles **40** starting from an *N*-tosyl protected precursor **39**, followed by deprotection (Scheme 14) [42]. The use of a protected precursor and optimized reaction conditions improved the yields of the final compounds. By varying the (hetero)aryl halides, this methodology allows for the creation of diverse meriolin derivatives, which have shown significant potential as multikinase inhibitors and apoptosis inducers. This versatile and efficient approach facilitates the synthesis of novel bioactive compounds, enabling further exploration of their chemical and biological properties. Recent studies on these novel meriolin derivatives have also revealed their cytotoxic and apoptotic effects, demonstrating their ability to induce mitochondrial apoptosis even in the presence of antiapoptotic Bcl-2 proteins. These findings position the derivatives as promising candidates for overcoming therapy resistance in cancer treatment, highlighting the therapeutic potential of these compounds [43,44].

**Scheme 15:**
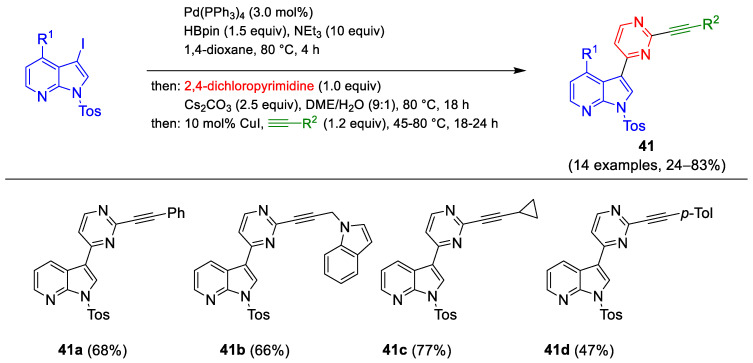
Consecutive three-component Masuda borylation–Suzuki arylation–Sonogashira alkynylation synthesis of 2-alkynyl-4-(7-azaindol-3-yl) pyrimidines **41** [45].

The extension of that approach by the same group adds an additional Sonogashira coupling step after the MBSC but without the final removal of the *N*-tosyl protection. This approach yields fluorescent 2-alkynyl-4-(7-azaindol-3-yl)pyrimidines **41**, with yields ranging from 24 to 83% (Scheme 15) [45]. The elimination of intermediate isolation through a single palladium catalyst system for all three catalytic steps marks a notable advance in operational efficiency. The resulting compounds display intense blue-to-green emissions under UV excitation, with their emission properties modulated by the types of substituents. Moreover, time-dependent density functional theory calculations confirmed their electronic structures and absorption bands, supporting the efficiency and robustness of the sequential synthesis. 

**Scheme 16:**
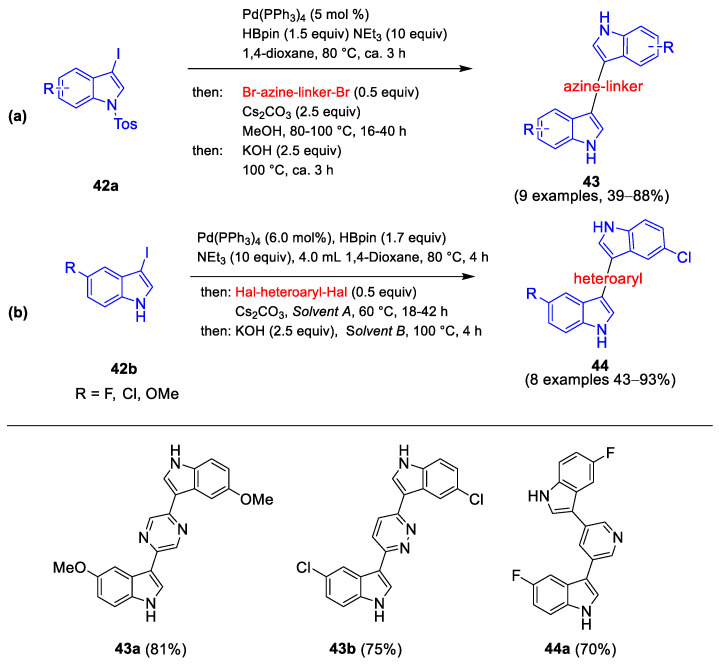
The MBSC route, involving deprotection as the final step, leads to bisindoles **43** and **44** [46].

MBSC sequences leading to symmetric bis-aryl substitution are among the simple, yet efficient examples of sequential reactions. Following this approach, Rehberg et al. reported the synthesis of bisindole derivatives of (di)azine- **43** or heteroaryl-building blocks **44** with potent antibacterial properties [46]. *N*-Tosyl-protected indoles **42** were used for borylation, as this improved the yield of the entire sequence, with the product deprotected in the final step (Scheme 16). Water as a co-solvent was crucial in optimizing reaction conditions to achieve high-purity bisindole derivatives. This methodology is particularly effective in synthesizing derivatives of natural compounds like alocasin A and hyrtinadine A, which exhibit significant antibacterial activity, especially against methicillin-resistant *Staphylococcus aureus* (MRSA). Notably, while unsubstituted bisindoles lacked antibacterial activity, the 5,5′-chloro derivatives showed high efficacy against MRSA and other Gram-positive pathogens, with minimal inhibitory concentrations between 0.20 and 0.78 μM. These compounds lead to bacterial membrane permeabilization, causing cellular efflux of low-molecular-weight molecules, which ultimately results in bacterial cell death.

Subsequent studies have focused on evaluating both the *N*-tosyl-protected and deprotected bisindoles regarding their antibacterial activities [47]. While the protected bisindoles did not show significant activity, their deprotected peers demonstrated potent MRSA inhibition by interacting with bacterial membrane components such as lipid II and phospholipids, leading to rapid membrane disruption and, consequently, bacterial cell death. Hence, the research experimentally proves the importance of the indole’s NH function in expressing antibacterial properties.

**Scheme 17:**
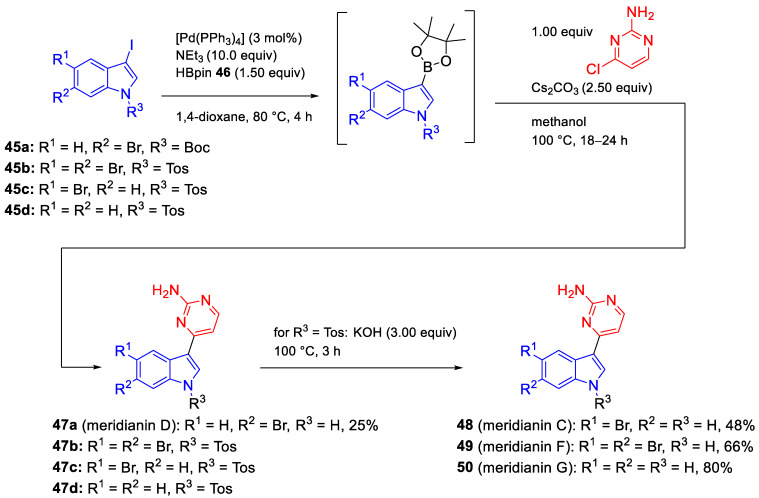
Synthesis of meridianins C **48**, D **47**, F **49**, and G **50** by MBSC sequence [48].

An MBSC sequence, starting with *N*-protected 3-iodoindole **45**, similar to the simple non-Suzuki-extended sequence discussed above, has been employed for synthesizing complex marine indole alkaloids such as meridianins D **47**, C **48**, F **49**, and G **50**, as well as scalaridine A [48]. These alkaloids exhibit significant biological activity, including potent protein kinase inhibition and cytotoxic effects, making them promising candidates for anticancer therapies. The initial borylation with pinacolborane **46** in the presence of [Pd(PPh_3_)_4_] is followed by Suzuki coupling with (di)azine halides, giving the desired alkaloids in moderate to excellent yields, maintaining operational simplicity and high functional group tolerance (Scheme 17).

### 2.2. Sequentially Pd-Catalyzed Processes Based upon Sonogashira–Hagihara Cross-Coupling

The well-known Sonogashira (or Sonogashira–Hagihara) reaction plays a frequent role in Pd-catalyzed reaction sequences. The resulting compounds often serve as valuable intermediates for subsequent transformations both within and beyond these sequences [49,50]. Some synthetic routes, including those toward natural products, become notably concise when they involve alkyne-based transformations [51]. Additionally, alkynes, as lightweight spacers that can be further functionalized, are also of significant interest in materials chemistry [52].

The mechanism of the Sonogashira reaction generally resembles that of other Pd-catalyzed cross-couplings and is intuitively well understood, even though certain mechanistic details remain unclear. Importantly, copper species may act as synergistic co-catalysts, but in this review, such variants are grouped together with the Pd-only cases. The advances in the applications of the Sonogashira reaction, as well as mechanistic insights, have been extensively covered in both classic and recent reviews. For this review, it is important to note that palladium *π* complexes with ethyne species, or formal palladium ethynides (acetylenides) that form toward the end of the catalytic cycle, are themselves reactive intermediates. These intermediates can undergo further transformations, such as participating in Heck-style reactions involving alkene insertion (particularly if *β*-elimination is possible) or alkyne insertion into the Pd-C bond. Such cases are of elevated interest, as they typically involve cascade or domino reactions, which offer significant advantages compared to reactions based on separate reaction steps.

**Scheme 18:**
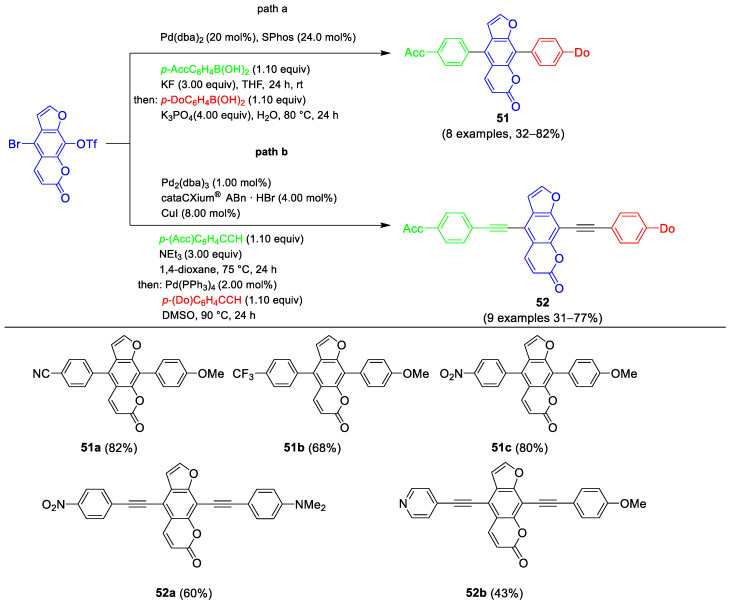
(Path a) Selective sequentially Pd-catalyzed Suzuki–Suzuki three-component synthesis of 5-donor/8-acceptor substituted psoralens **51**; (Path b) similar Sonogashira–Sonogashira three-component synthesis of alkynyl-expanded 5-donor–8-acceptor-substituted psoralens **52** [53].

**Scheme 19:**
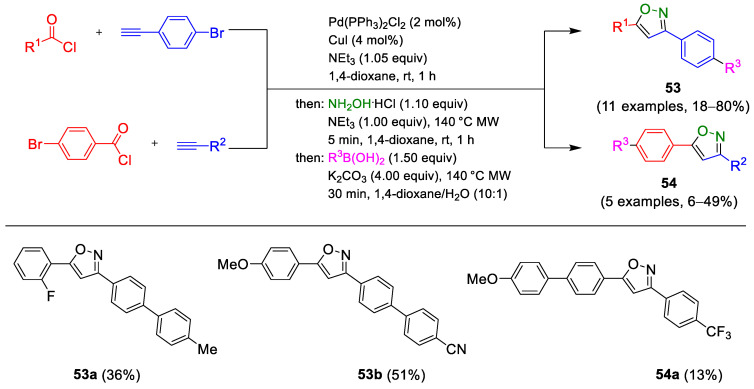
Consecutive coupling–condensation–coupling synthesis of 3-biarylsubstituted isoxazoles **53** and 5-biarylsubstituted isoxazoles **54** [55].

Geenen et al. reported the synthesis of psoralen-based cruciforms **51** and **52** with asymmetric donor–acceptor functionalization [53,54]. This case exemplifies the utility of simple “homo-sequences” like Suzuki–Suzuki and Sonogashira–Sonogashira reactions, thus serving as a suitable transition between the sections in this review. The one-pot approach enables higher yields compared to the classical consecutive approach, reducing both catalyst consumption and reaction time (Scheme 18). The tunable asymmetric donor–acceptor molecular design of heterocycles is frequently employed when optimizing photophysical properties, including solvatochromism, acidochromism, and aggregation-induced emission. These characteristics render such heterocyclic structures promising candidates for applications in molecular photonics and theranostics. In another advancement, Deden et al. presented an interesting example of a sequence with an alkyne intermediary leading to novel 3- and 5-biaryl-substituted isoxazoles **53** and **54** via a four-component synthesis [55]. Inspired by their previous work on the four-component synthesis of biaryl-substituted pyrazoles, the authors adapted a sequentially Pd-catalyzed process for the multicomponent reaction (MCR) formation of biarylated isoxazoles [56]. This microwave-assisted synthesis concatenates an acyl–Sonogashira coupling, cyclocondensation with hydroxylamine, and Suzuki coupling in a one-pot fashion (Scheme 19). The Pd catalyst was employed sequentially without further addition of catalyst loading, achieving high catalyst usage efficiency. The synthesized biaryl-substituted isoxazoles exhibited remarkable photophysical properties, including high fluorescence quantum yields up to *Φ*_F_ = 0.86 and large Stokes shifts up to 10,000 cm^−1^. These photonic characteristics were rationalized using density functional theory (DFT) and time-dependent DFT (TD-DFT) calculations.

**Scheme 20:**
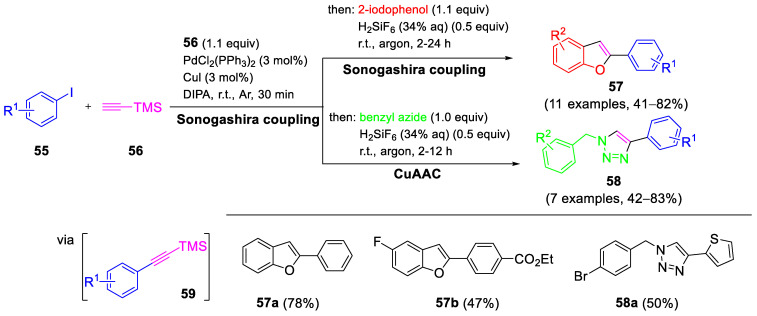
One-pot synthesis of 2-arylbenzofuran derivatives **57** via Sonogashira–Sonogashira and 4-aryl-1-benzyl-1*H*-1,2,3-triazoles **58** derivatives via Sonogashira–CuAAC sequences [57].

Sinai and colleagues further extended the application of sequential Sonogashira reactions by developing a one-pot synthesis that combines Sonogashira coupling and click chemistry for efficient construction of heterocycles such as benzofurans **57** and triazoles **58** [57]. The process begins with the Pd,Cu-catalyzed Sonogashira coupling of aryl halides (aryl iodides) **55** with ethynyltrimethylsilane **56**, forming the corresponding trimethylsilyl-protected alkynes (Scheme 20). These intermediates undergo in situ desilylation using hexafluorosilicic acid, generating intermediary acetylenes **59**. The latter can undergo intermolecular heterocyclization via intermolecular hydroalkoxylation to yield benzofuran derivatives **57**. Alternatively, the acetylene moiety can be subjected to a copper-catalyzed azide–alkyne cycloaddition (CuAAC) to yield 1,4-disubstituted triazoles **58**. Both processes highlight the concise and efficient approach to sequences leading to annulated heterocycles via acetylenes under mild conditions. 

**Scheme 21:**
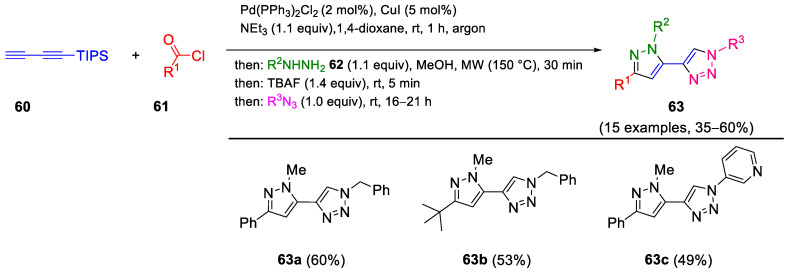
Consecutive alkynylation–cyclocondensation–desilylation–CuAAC (ACDC) sequence for the four-component synthesis of 4-pyrazolyl-1,2,3-triazoles **63** [58].

Niesobski et al. developed a one-pot, four-step, Pd-Cu catalyzed strategy for synthesizing 4-pyrazolyl-1,2,3-triazoles **63** through a consecutive acyl–Sonogashira alkynylation–cyclocondensation–desilylation–CuAAC (ACDC) sequence [58]. This work is part of a broader effort to advance heterocyclic synthesis via sequential catalysis. The strategy integrates sequential Pd-Cu dual catalysis and employs TIPS–butadiyne **60** as a central four-carbon building block, which undergoes Sonogashira alkynylation with aroyl chlorides **61**, followed by a cyclocondensation reaction with hydrazines **62**. After desilylation, the final Cu-catalyzed azide–alkyne cycloaddition (CuAAC) step efficiently generates the desired triazole derivatives **63** (Scheme 21). This methodology is notable for its operational simplicity and high regioselectivity achieved across various substrates, making it an efficient approach for constructing complex biheteroaryls.

The same team further expanded their work on Pd/Cu-catalyzed multicomponent reactions by developing a novel and facile one-pot synthesis of diaryl 1,2-diketones **68** and quinoxalines **69** [59]. 

**Scheme 22:**
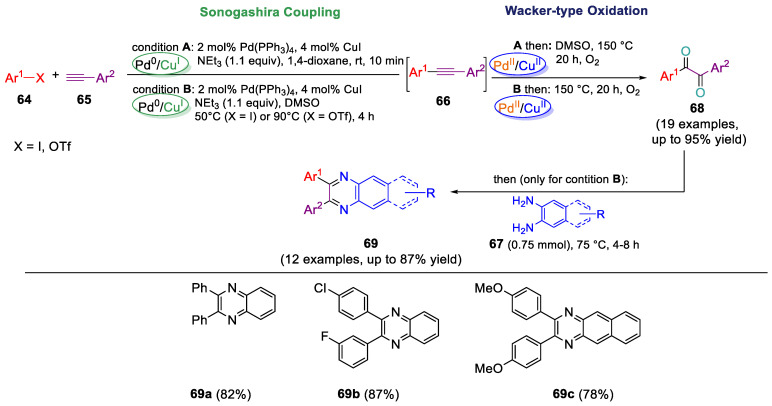
One-pot sequential Pd/Cu-catalyzed alkynylation and oxidation for the synthesis of 1,2-diketones **68** and quinoxalines **69** [59].

This method integrates two distinct Pd/Cu-catalyzed steps: it initiates with a Sonogashira coupling of aryl iodides or triflates **64**, with terminal alkynes **65** to form diarylacetylenes **66**, followed by a Wacker-type oxidation using DMSO and oxygen as dual oxidants (Scheme 22). The process efficiently produces symmetrically and unsymmetrically substituted diketones in high yields, with excellent tolerance for a wide range of functional groups. The diketones are then directly converted into quinoxalines **69** through a subsequent cyclocondensation step by adding 1,2-diaminobenzene **67** derivatives in the same reaction vessel. This efficient and adaptable approach not only streamlines the synthesis of benzils but also serves as a foundation for other consecutive multicomponent syntheses of heterocycles, with ongoing mechanistic studies of the oxidation step further broadening its potential applications.

**Scheme 23:**
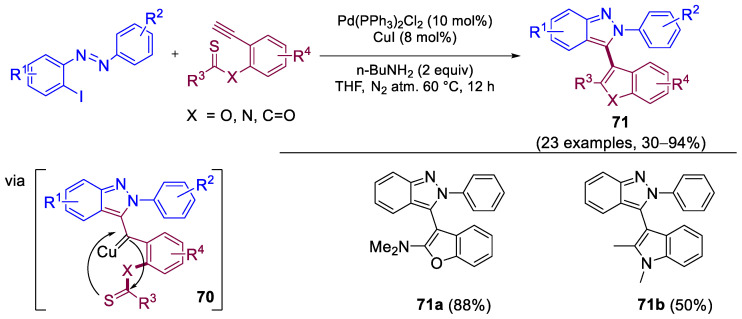
Synthesis of indazole-containing biheteroaryls **71** via Sonogashira coupling–azaenyne cycloisomerization–Barton–Kellogg reaction cascade [60].

Ding et al. introduced an innovative approach to the synthesis of indazole-containing biheteroaryls **71** via a cascade Sonogashira coupling–intermolecular azaenyne cyclization–intermolecular Barton–Kellogg-type reaction [60]. The Sonogashira coupling can seemingly be separated into a single sub-step, which is why the reaction is reviewed as sequential (Scheme 23). Mechanistically, the reaction could proceed without copper, although copper’s catalytic assistance, or even its primary role, was observed to be important for the smooth progression of each reaction step. This novel strategy leverages a metal carbene intermediate **70** to construct two heterocycles, employing an acetylene moiety as a one-carbon source for two different five-membered heterocycles. The scalable protocol was tested and successfully applied to synthesize a wide range of substituted indazole-containing biheteroaryls under mild reaction conditions.

**Scheme 24:**
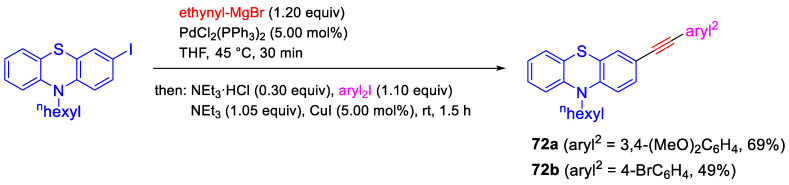
Synthesis of arylethynyl phenothiazine derivatives **72a** and **72b** via the Kumada–Sonogashira methodology [62].

An interesting sequence involving two non-classical variants of the Sonogashira coupling, followed by cyclocondensation to yield substituted pyrazoles or pyrimidines, was reported by Götzinger et al. [61]. The first step employs a Kumada–Sonogashira reaction, based on a previously developed methodology [62], and is illustrated with isolated intermediate **72** in Scheme 24.

**Scheme 25:**
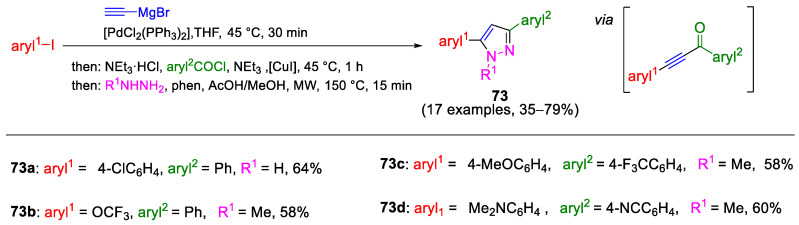
Sequentially Pd-catalyzed Kumada–Sonogashira–acyl–Sonogashira–cyclocondensation sequence used for the synthesis of trisubstituted pyrazoles **73** in a consecutive four-component fashion [61].

**Scheme 26:**
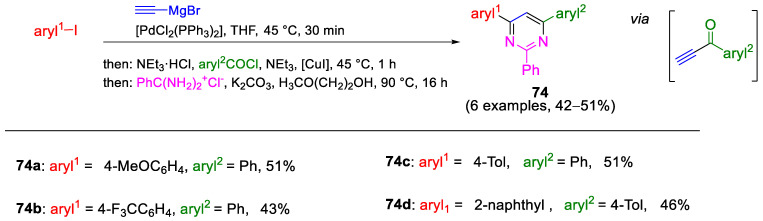
Sequentially Pd-catalyzed Negishi–Sonogashira–acyl–Sonogashira–cyclocondensation sequence used for the synthesis of pyrimidines **74** in a consecutive four-component fashion [61].

This methodology was successfully extended toward the synthesis of heterocycles via alkynones, which are valuable heterocycle precursors. Ethynylmagnesium bromide is used in the first Kumada–Sonogashira step to produce a monosubstituted acetylene, which undergoes an acyl–Sonogashira reaction, yielding a disubstituted acetylene in the form of an alkynone (Scheme 25 and Scheme 26). Finally, the alkynones are cyclocondensed with N-monosubstituted hydrazines or C-monosubstituted diaminomethanes to yield pyrazoles **73** and pyrimidines **74**, featuring up to three distinct substituents.

Several of the obtained pyrazoles, bearing both electron-donating and electron-accepting substituents, exhibit fluorescence with notable positive solvochromism. The emission maxima span an overall range from 363 nm (cyclohexane) to 595 nm (acetonitrile), with a dipole moment change of Δ*μ* = 46 D from the ground to the excited state. (TD)DFT calculations provide insights into the electronic structure and significant charge-transfer character of the lowest energy absorption bands.

### 2.3. Sequentially Pd-Catalyzed Processes Based upon Heck Reaction

The Misoroki–Heck (further Heck) reaction [63], a prominent alkenylation reaction, has been widely adapted in sequentially Pd-catalyzed one-pot syntheses, offering a versatile platform for the synthesis of complex heterocycles. This section delves into various studies that utilize the Heck reaction, often in combination with other palladium-catalyzed processes, to streamline the formation of intricate molecular architectures. From tandem reactions to innovative cascade processes, these methodologies highlight the power of the Heck reaction in enabling efficient, regioselective, and sustainable syntheses, particularly in the realm of heterocyclic chemistry [64].

**Scheme 27:**
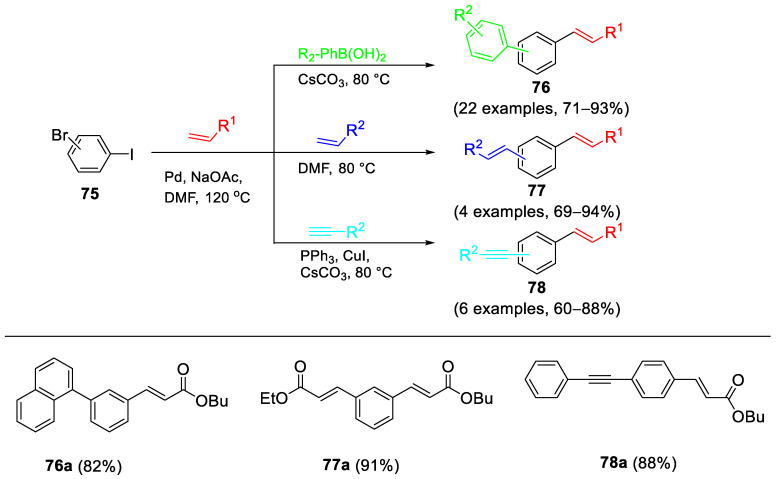
Palladium-catalyzed one-pot sequential Heck–Suzuki, Heck–Heck, and Heck–Sonogashira couplings of aryl dihalides **75** yielding unsymmetrically substituted arenes **76**, **77**, and **78** [65].

X. Zhang, A. Liu, and W. Chen focused on demonstrating the utility of straightforward “basic” reaction combinations involving the pairing of a Heck reaction with other Pd-catalyzed reactions like Suzuki or Sonogashira or another Heck reaction using bromo-iodo-arenes **75** in a one-pot sequence [65]. By employing Pd-NHC complexes as catalysts, the authors achieve regioselective C–C bond formation by exploiting the different reactivity of the halogens, producing unsymmetrically substituted arenes **76**, **77**, and **78** with excellent efficiency and overall yields (Scheme 27). The methodology also highlights the efficiency of Pd-NHC catalysis in multistep organic transformations. 

The alkene moiety, acting as both an assembly point and a synthon for an ethylene bridge between aryl groups, allows for the use of Heck-based sequences for concise transformations.

**Scheme 28:**
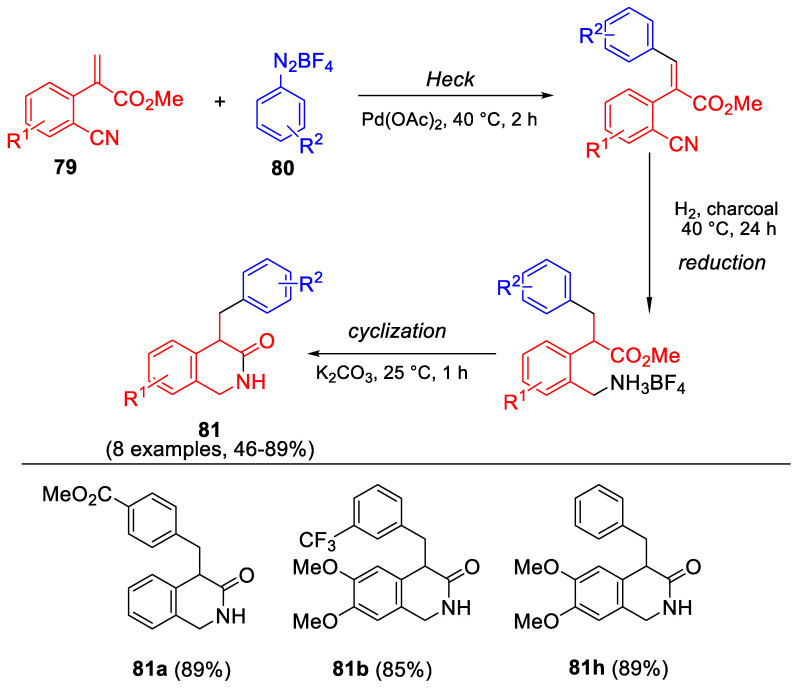
Heck–reduction–cyclization sequences (HRC) for the preparation of 4-benzyl-1,2-dihydroisoquinolin-3-ones **81** [66].

In a related study, Felpin and coworkers employed a Heck–reduction–cyclization (HRC) sequence for synthesizing 4-benzyl-1,2-dihydroisoquinolin-3-ones **81** [66], which is an evolution of their earlier work on oxindole and 2-quinolone synthesis [67,68,69]. The approach utilizes a multifunctional palladium catalyst that initially operates in a homogeneous phase for the Heck coupling of 2-(2-cyanoaryl)acrylates **79** with diazonium salts **80** (Scheme 28). The catalyst is then converted in situ to a heterogeneous Pd/C catalyst for the subsequent reduction and cyclization sub-steps. This strategy offers the efficient formation of complex heterocyclic structures by elegantly using two “masked functionalities” actualized through reduction under sustainable conditions, with catalyst recyclability as an added advantage. 

**Scheme 29:**
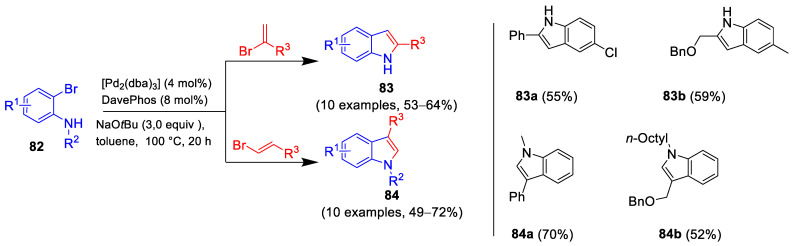
The reaction of alkenyl bromides with *o*-bromoanilines **82** for the synthesis of 1*H*-indoles **83** and indoles **84** [70].

A notable example of a selective cascade reaction leading to indoles **83** and **84**, starting from o-bromoanilines **82** and alkenyl halides, was demonstrated by Barluenga et al. [70]. The cascade involves a Heck reaction followed by an intermolecular Buchwald–Hartwig amination (Scheme 29).

The desired formation of indoles is observed in remarkably good yield, considering the possibility of alternative reactions, particularly homocouplings. While the individual steps of the reaction are not separated, there is potential for further yield improvement if carried out as a sequential process. According to the authors, this is the first reported example of alkenyl amination reactions participating in Pd-catalyzed cascade sequences. Their study confirms the expected relative reactivity trend in amination, with the reactivity order decreasing as follows: alkenyl bromides, aryl bromides, alkenyl chlorides, and aryl chlorides. Under optimized conditions using [Pd_2_(dba)_3_]/DavePhos (dba = dibenzylideneacetone, DavePhos = 2-dicyclohexylphosphano-2′-*N*,*N*-dimethylaminobiphenyl), NaO*t*Bu, and toluene at 100 °C, the reaction achieves enhanced chemoselectivity and versatility, effectively synthesizing indoles with a broad range of functional groups.

**Scheme 30:**
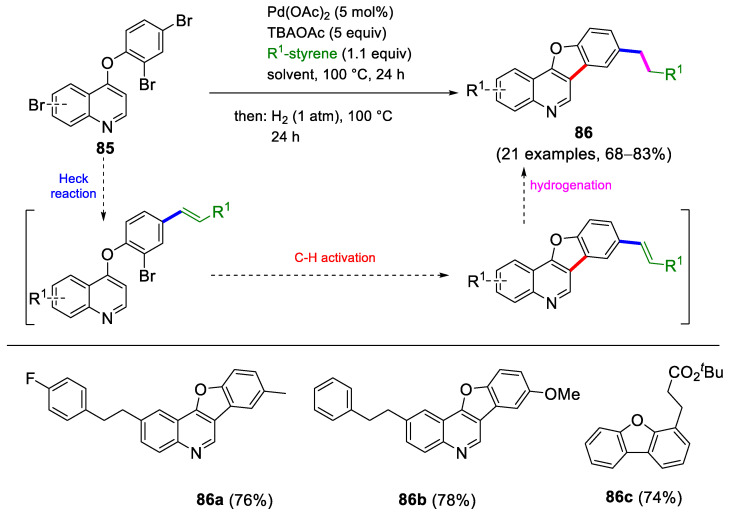
One-pot Heck-directed arylation–hydrogenation sequence for the synthesis of multicyclic alkylated heteroarenes **86** [5].

In a significant development, Kehoe and colleagues introduced a novel, phosphane-free, inorganic base-free, one-pot reaction sequence that combines Heck olefination, direct arylation, and hydrogenation [5]. Similar to previous cases, the alkene moiety functions as a precursor to an ethylene bridge. The palladium catalyst facilitates all three key transformations: olefination, C–H activation, and hydrogenation (Scheme 30). 

This method enables the efficient synthesis of complex, medicinally relevant heterocycles, particularly quinolines **86**, while adhering to green chemistry principles and significantly enhancing both mass productivity and solvent efficiency. The use of tetrabutylammonium acetate (TBAOAc) as an ionic liquid and base simplifies the reaction conditions and broadens the substrate scope to include pyridines and simple aryl substrates **85**. Additionally, the study explored deuterium incorporation using the COware apparatus, further expanding the method’s applicability in medicinal chemistry.

**Scheme 31:**
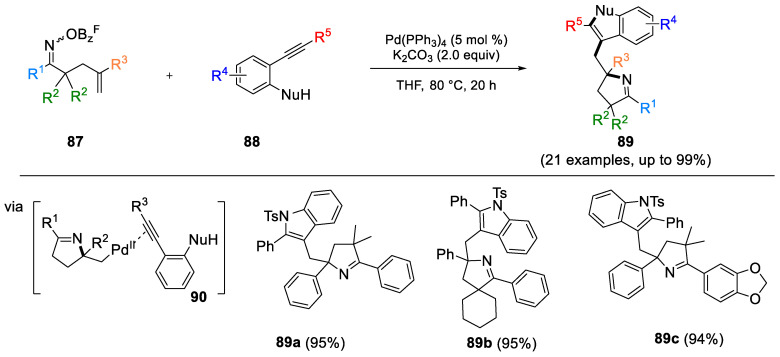
Nasaraka–Heck–Cacchi cascade involving alkene-tethered oxime esters and *o*-alkynyl anilines to yield-(dihydro-2*H*-pyrrol-2-ylmethyl)indoles **89** [71].

**Scheme 32:**
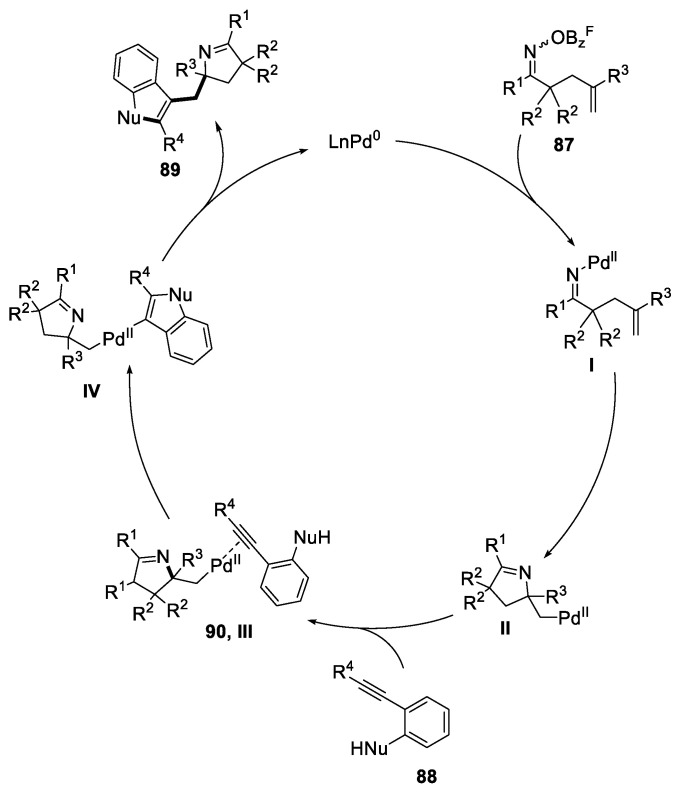
Proposed mechanism for palladium-catalyzed domino Narasaka–Heck cyclization via oxime ester activation and Cacchi reaction [71].

An example of an advanced case is the use of the Narasaka–Heck cascade reaction, developed for the synthesis of a novel class of unsymmetrical alkyl-linked bis-heterocycles by Arora et al. [71]. The cascade begins with Pd(0)-catalyzed iminopalladation of alkene-tethered oxime esters **87**, forming the 3,4-dihydro-2*H*-pyrrole ring **89** in the course of the Narasaka–Heck reaction (Scheme 31). The palladium intermediate **90** formed in this step is coordinated by an alkyne **88**, initiating a Cacchi reaction with *o*-alkynyl anilines, which leads to the fusion of the previously formed pyrrole ring with newly formed indoles. This cascade reaction, featuring the formation of three new covalent bonds and two rings, achieves excellent overall yields (up to 99%) and demonstrates both broad functional group tolerance and high efficiency, even with sterically hindered substrates. Mechanistic studies confirmed a two-electron migratory insertion process, which is easily scalable to millimolar quantities while maintaining high efficiency (Scheme 32). Although this is not strictly a sequential reaction, its stepwise nature and the potential utility of a generalized sequential approach justified its inclusion in this review. The demonstrated method broadens the scope of the Narasaka–Heck reaction, providing a valuable tool for synthesizing complex heterocyclic scaffolds.

**Scheme 33:**
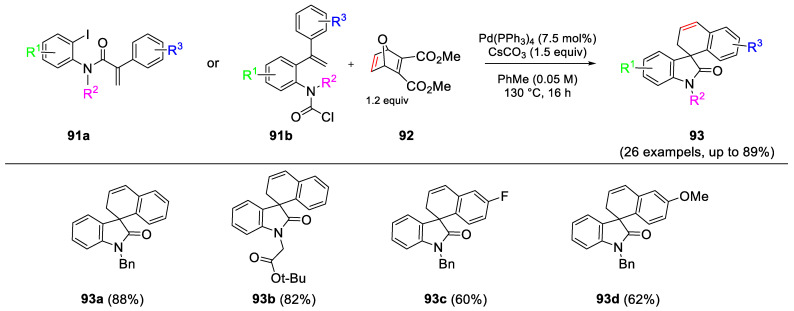
Palladium-catalyzed spiro-cyclization of aryl iodides **91** and carbamoyl chlorides **92** to form spiro-oxindoles **93** [72].

Another cascade reaction showcasing the capabilities of one-pot reactions to form synthetically challenging spiro-compounds **93** was presented by Abel-Snape et al., involving two formal alkene insertions (i.e., Heck-type transformations) into organopalladium intermediates during the course of the reaction [72]. The synthesis of spiro-oxindoles begins with the formation of the indolone cycle with a methylpalladium pendant group, similar to the previous sequence initiated by the Narasaka–Heck reaction (Scheme 33). 

**Scheme 34:**
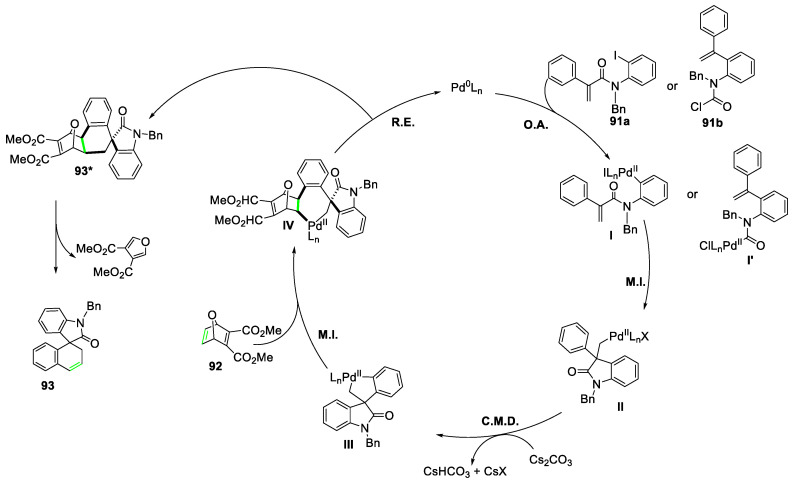
Proposed mechanism for palladium-catalyzed C–H activation and spiro-oxindole formation via acetylene insertion [72].

The palladium intermediate converts to a palladacycle via directed C–H activation. The next key sub-step involves the diastereoselective alkene insertion of an unsaturated oxabicycle—specifically, a 1,4-oxo-cyclohexadiene derivative **92** acting as a masked acetylene in the form of a labile Diels–Alder product—into the palladacycle. The process is finalized by a retro-Diels–Alder reaction that releases the spiro-oxindole product and regenerates the palladium catalyst. The authors provide mechanistic evidence supporting this reaction pathway, indicating that C–H activation precedes oxabicycle insertion, facilitating the efficient formation of spiro-oxindole structures (Scheme 34).

These spiro-oxindoles, characterized by unique structural features, also facilitate transformations that were previously unattainable, making this method highly versatile and effective for producing a diverse range of spiro-oxindole derivatives with good yields.

### 2.4. Sequentially Pd-Catalyzed Processes Based upon Hydroamination 

A frequent and recognizable two-reaction combination among the described Pd-catalyzed sequences is the formation of an alkyne, typically supplied by a Sonogashira reaction, followed by intermolecular hydroamination to yield an annulated aza-arene. The Cacchi reaction is one of the well-known named reactions based on this route [73,74]. Hydroamination is a key process for the efficient C–N bond formation, essential for synthesizing nitrogen-containing heterocycles widely used in pharmaceuticals and materials science. Consequently, hydroamination is typically not the initial reaction in the sequence but plays an important role in streamlining the construction of complex aza-heterocycles [75].

The choice of reaction conditions, particularly the nature of alkynes and amines used, is critical in controlling both the regioselectivity and yield. Pd catalysis in this context allows for fine-tuning these parameters, ensuring high selectivity during the hydroamination step. Additionally, hydroamination readily integrates with other elementary steps, such as oxidative addition or reductive elimination, enabling the formation of complex heterocyclic systems in a single pot [76].

**Scheme 35:**
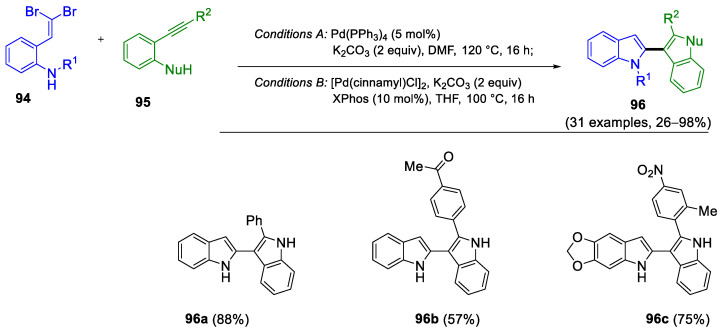
Synthesis of unsymmetrically linked bis-heterocycles via a cascade process utilizing two slightly different Cacchi domino reactions [77].

**Scheme 36:**
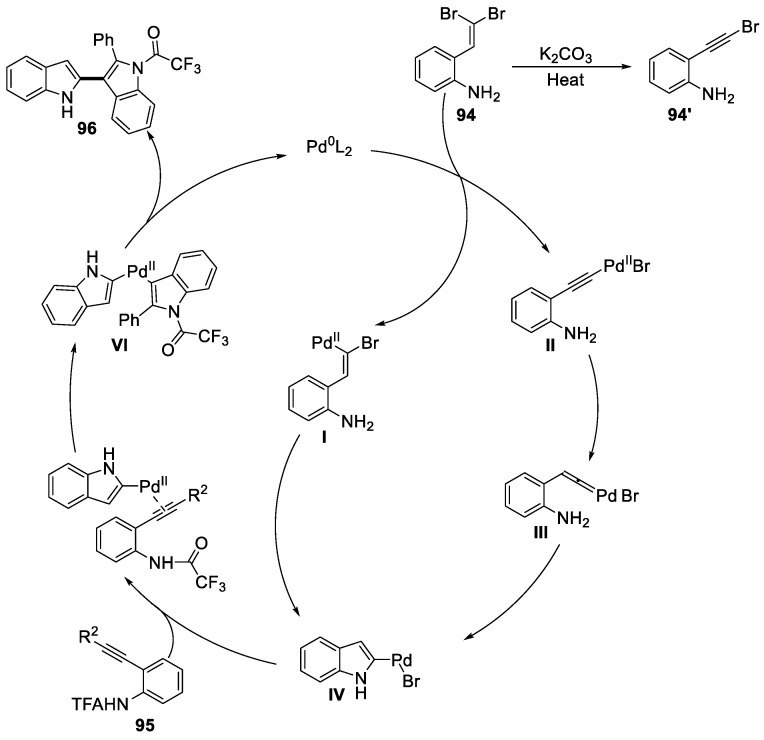
Mechanistic pathway of palladium-catalyzed bis-heterocycle synthesis via sequential C–N coupling and Cacchi reaction [77].

Another study introduces a new approach for synthesizing unsymmetrically linked bis-heterocycles in a cascade, relying on two Cacchi domino reactions (Scheme 35), where the two Cacchi reactions are slightly different [77]. The whole sequence could be described as alkene dehydrobromination–Cacchi reaction–Cacchi reaction with concomitant coupling. This approach enables the creation of complex biheterocyclic structures, such as 1,4-disubstituted biaryls, through alternative mechanisms involving different types of intermediates (Scheme 36). The study also examines the electronic and steric properties of the synthesized compounds, emphasizing their potential applications in medicinal chemistry and materials science.

**Scheme 37:**
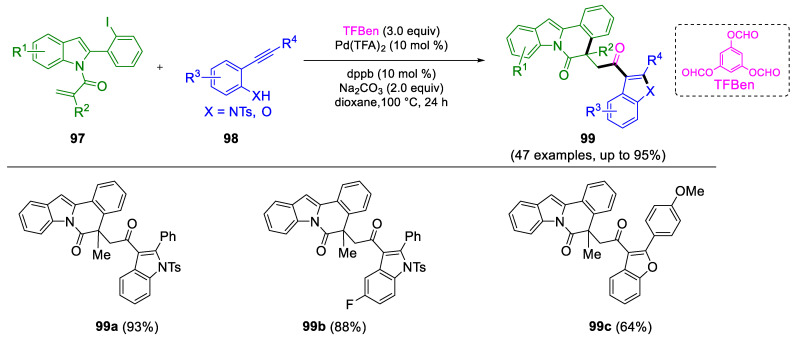
Palladium-catalyzed domino carbonylative cyclization for the synthesis of indolo[2,1-*a*]isoquinoline–indole and indolo[2,1-*a*]isoquinoline–dihydrobenzofuran derivatives **99** [78].

**Scheme 38:**
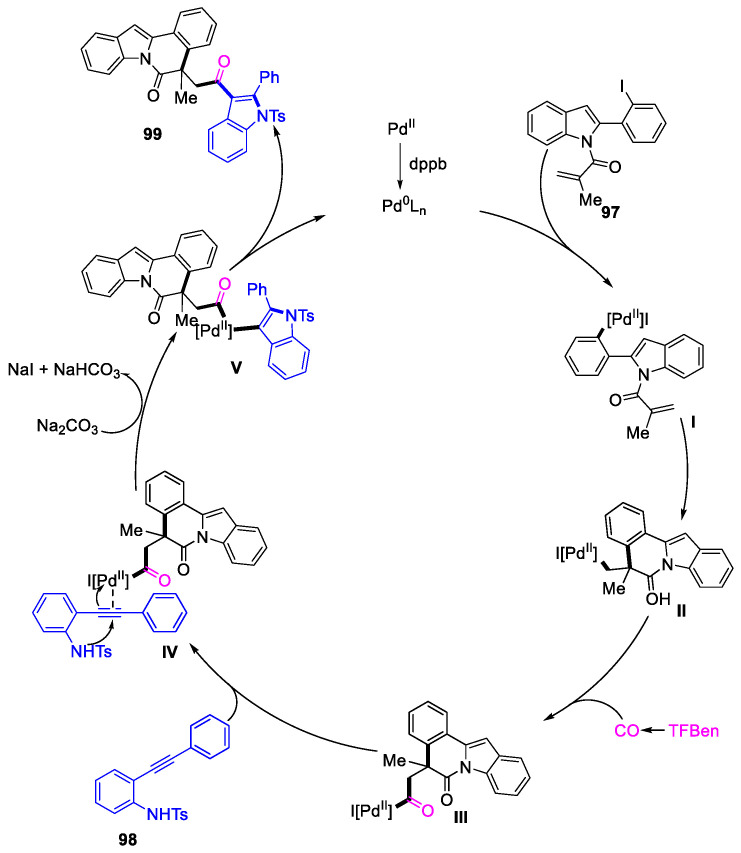
Proposed mechanism for palladium-catalyzed domino carbonylative cyclization involving CO-linked bis-heterocycle formation [78].

Another innovative method for constructing functionalized heterocycles has been developed through a palladium-catalyzed carbonylative cyclization cascade [78]. This reaction proceeds via sequential coupling of aryl halides with isocyanides, followed by carbonylation using benzene-1,3,5-triyl triformate (TFBen) as the CO source, and intramolecular cyclization (the final step is formally being an intramolecular Suzuki coupling). Notably, this cascade enables the consecutive formation of three C–C bonds and one C–X bond (where X can be N or O), leading to the synthesis of biologically relevant indolo[2,1-a]isoquinoline–indole and indolo[2,1-*a*]isoquinoline–dihydrobenzofuran **99** derivatives in high yields (Scheme 37 and Scheme 38). This approach proves to be highly effective for synthesizing diverse heterocyclic compounds, demonstrating broad substrate compatibility.

**Scheme 39:**
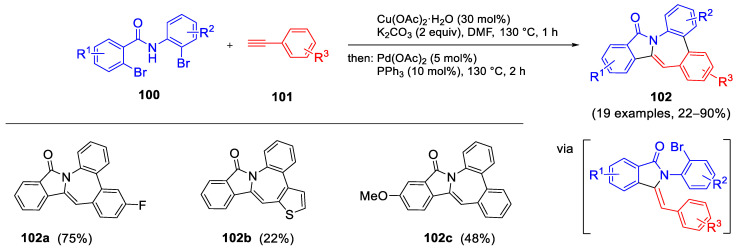
Sequential copper- and palladium-catalyzed one-pot synthesis of azepino-fused isoindolinones **102** from substituted 2-bromo-*N*-(2-bromoaryl)benzamides **100** and arylacetylenes **101** [79].

Continuing this line of research, Saini et al. developed a sequential one-pot method, contrasting with the cascade approach in the previous case, for synthesizing azepino-fused isoindolinones 102 [79]. The method begins with copper-catalyzed Sonogashira coupling to form the initial carbon–carbon bond, followed by hydroamination to introduce a nitrogen atom, and concludes with palladium-catalyzed directed arylation to complete the fused ring system (Scheme 39). This sequence efficiently forms two carbon–carbon bonds and one carbon–nitrogen bond, generating azepino-fused isoindolinones with high regioselectivity and functional group tolerance under mild conditions, yielding a diverse array of derivatives.

**Scheme 40:**
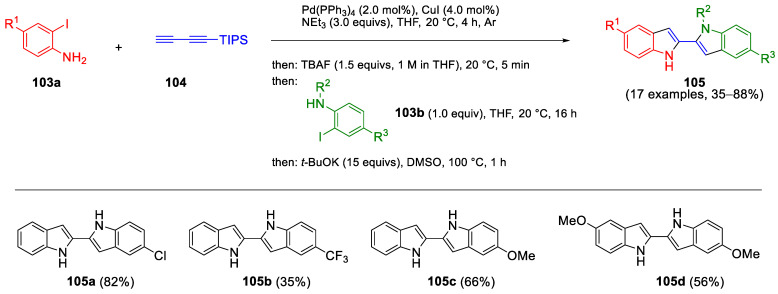
Consecutive three-component alkynylation–desilylation–alkynylation–cycloisomerization (ADAC) synthesis of unsymmetrically substituted 2,2′-bi-indolyls **105** [80].

The ADAC process, a sequentially Pd/Cu-catalyzed one-pot synthesis, where copper appears to play an assisting role, exemplifies an advanced method for constructing heterocycles such as 2,2′-bi-indolyls **105** [80]. This concise sequence begins with the Sonogashira alkynylation of *ortho*-iodoanilines **103** using TIPS–butadiyne **104** as a four-carbon building block, followed by Cacchi hydroamination, desilylation, subsequent arylation, and intramolecular hydroamination (Scheme 40). The process efficiently forms four new bonds and two rings within a single reaction vessel, demonstrating its applicability to a variety of functionalized substrates. The ability to generate complex bi-indolyl frameworks in high yields with diverse substitution patterns makes this method particularly valuable for synthesizing biologically active compounds, such as tjipanazole I.

**Scheme 41:**
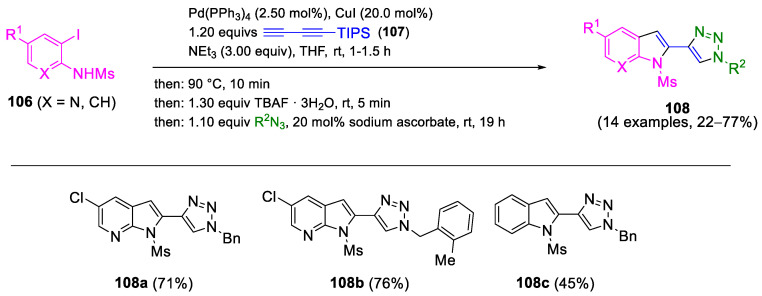
One-pot coupling–cyclization–desilylation–CuAAC synthesis of 7-azaindoles **108a**,**b** and indole **108c** [81].

A novel approach for synthesizing 1*H*-1,2,3-triazol-4-yl-1*H*-pyrrolo[2,3-*b*]pyridines **108** has been realized through a one-pot, Pd/Cu-catalyzed process that elegantly integrates coupling, cyclization, and desilylation reactions, executed in a three-component fashion [81]. This process crucially relies on the Sonogashira coupling and Cacchi reaction sub-sequence. The reaction sequence begins with Pd-catalyzed alkynylation of 3-halo-2-aminopyridines **106** using tri(isopropyl)silyl butadiyne **107** (TIPS–butadiyne), followed by indole formation via Cacchi hydroamination (Scheme 41). The sequence then proceeds with a desilylation and concludes with triazole ring formation via Cu-catalyzed azide-alkyne cycloaddition (CuAAC). This method can be further expanded to a four-component process by including the in situ azid generation from alkyl halides. This approach efficiently generates triazole-substituted azaindoles, leveraging the synergistic Pd and Cu catalysis to deliver high yields and broad substrate compatibility, making it a valuable tool for synthesizing bioactive molecules.

### 2.5. Sequentially Pd-Catalyzed Processes Based upon Directed C-H Activation 

The activation of inert bonds remains a pivotal goal in synthetic organic chemistry. Directed C–H activation [82,83] is a special case that generally encompasses common reactions such as Heck coupling and more complex processes, including alkene C–H activation, which is sometimes called the “Holy Grail” due to its synthetic complexity. In this review, the focus will be primarily on the directed activation of C_Ar_–H bonds, i.e., the functionalization of aromatic species, as palladium catalysis has proven itself to be an invaluable strategic tool in organic synthesis. In the context of reaction sequences, organopalladium intermediates formed in prior sub-steps can be particularly efficiently involved in intermolecular heterocyclizations with ring closures at non-substituted C_Ar_–H positions. This section explores various methodologies that leverage directed C–H activation, often in combination with other palladium-catalyzed transformations, to synthesize diverse heterocycles with potential applications in pharmaceuticals and materials science [84].

**Scheme 42:**
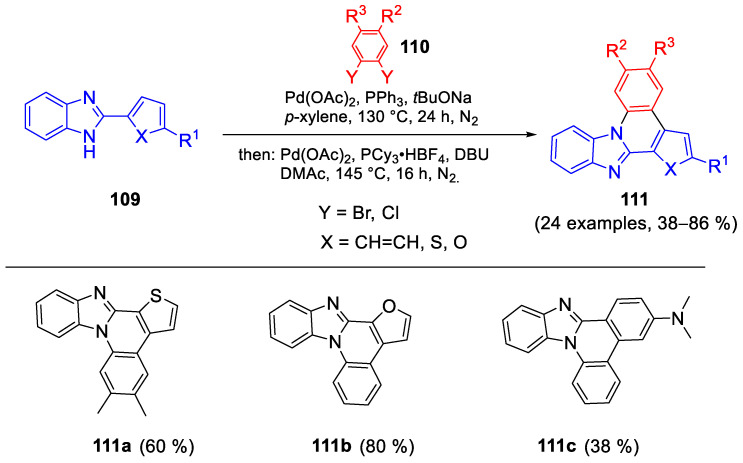
Synthesis of benzimidazo[1,2-*f*]phenanthridines **111** by a Pd-catalyzed sequential N-arylation and intramolecular C–H arylation [85].

Liu et al. introduced a straightforward one-pot strategy for synthesizing benzimidazo[1,2-*f*]phenanthridines **111** through a sequence of arylation and intramolecular coupling [85]. The synthesis begins with the N-arylation of 2-phenyl-1*H*-benzo[*d*]imidazoles **109** with 1,2-dibromobenzenes **110**, followed by intermolecular C–H arylation, leading to a five-ring fused aromatic system. The optimized conditions involved PPh_3_ as a ligand and *t*BuONa as a base in *p*-xylene at 130 °C for 24 h, followed by the second step at 145 °C (Scheme 42). The method tolerates a wide range of functional groups, enabling the formation of polysubstituted phenanthridine derivatives in yields ranging from 38 to 86%. While substrates with H or Me substituents at the *para* position of the phenyl ring of 2-phenyl-1*H*-benzo[*d*]imidazoles provide high product yields, the presence of mesomeric electron donors such as OMe, NMe_2_, and Cl tends to be less favorable. 

**Scheme 43:**
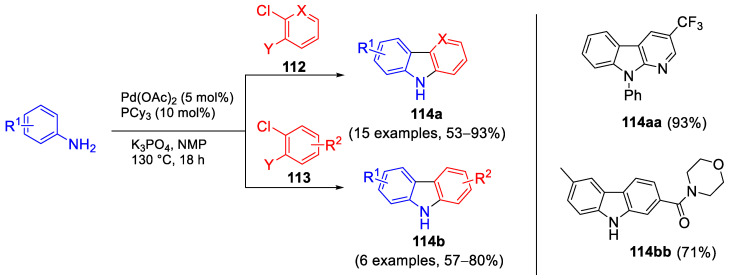
Synthesis of carbazole derivatives **114** from unsubstituted 1,2-dihaloarenes **112** and substituted 1,2-dihaloarenes **113** [86].

A similar example of two sub-step ring construction involving an *ortho*-substituted arene was reported by Ackermann et al. for synthesizing functionalized indoles or carbazoles **114** via a palladium-catalyzed sequence comprising intermolecular amination and intramolecular directed C–H arylation [86]. The described protocol is highly regioselective when using simple, cost-effective 1,2-dichloroarenes **112** or **113** as electrophiles (Scheme 43). Optimization studies revealed that polar aprotic solvents and weaker inorganic bases significantly enhance catalytic performance, suggesting potential for further cost-effective improvement.

**Scheme 44:**
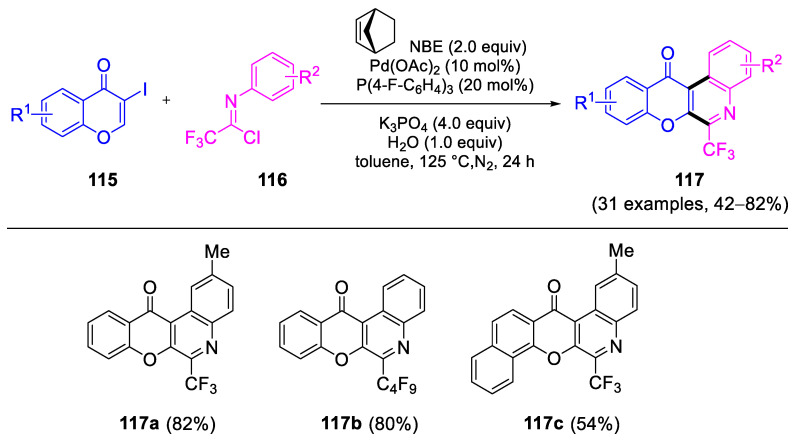
Pd-catalyzed norbornene-mediated anellation of 3-iodochromones **115** with trifluoroacetimidoyl chlorides **116** to yield trifluoromethyl-substituted chromeno[2,3-*c*]quinolin-12-ones **117** [87].

**Scheme 45:**
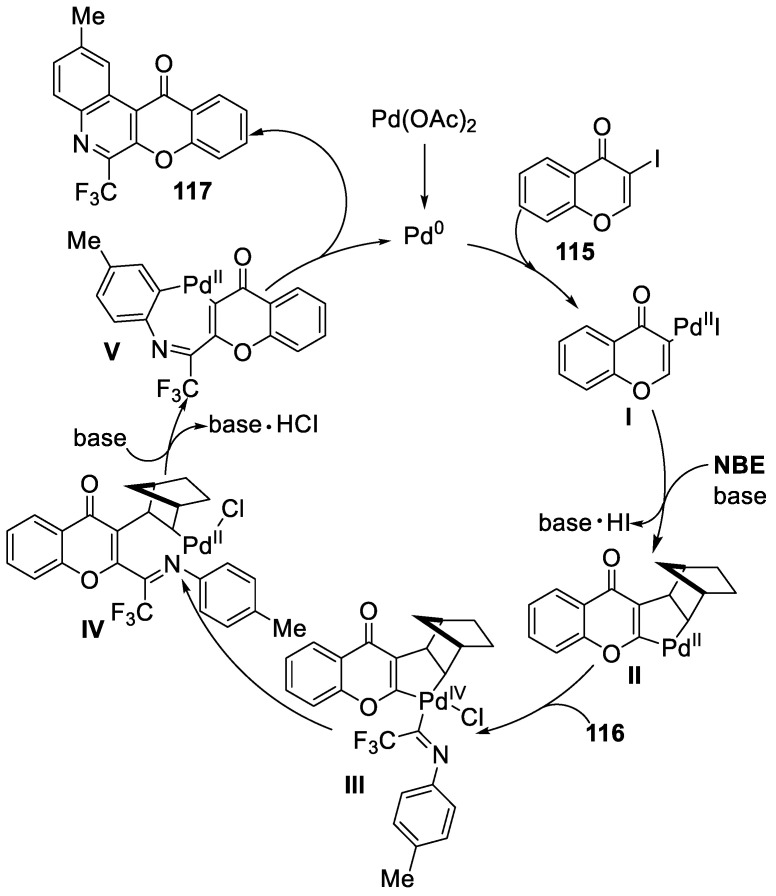
Proposed mechanism for palladium-catalyzed norbornene-mediated dehydrogenative anellation for the synthesis of trifluoromethyl-substituted chromeno[2,3-*c*]quinolin-12-ones **117** [87].

Expanding the scope of Pd-catalyzed C–H activation, Tang et al. introduced a method for constructing trifluoromethyl-substituted chromeno[2,3-*c*]quinolin-12-ones **117** via a palladium-catalyzed norbornene-mediated dehydrogenative anellation [87]. This is a cascade rather than a sequential process and is included here to demonstrate the power of one-pot methodologies. The strategy relies on palladium/norbornene (Pd/NBE) cooperative catalysis, often referred to as a Catellani-type reaction (i.e., *ipso*-functionalization of aryl iodides) (Scheme 44). The latter efficiently couples 3-iodochromones **115** with trifluoroacetimidoyl chlorides **116**, followed by an intermolecular ring closure via directed C–H activation to form complex trifluoromethylchromone derivatives **117** (Scheme 45). The conversion, which features a broad substrate scope and good yields, underscores the utility of Pd/NBE catalysis in the construction of fluorinated N-heterocycles and, in a broader sense, the potential of reaction sequences using the Catellani-type reaction for creating annulated heterocyclic systems.

**Scheme 46:**
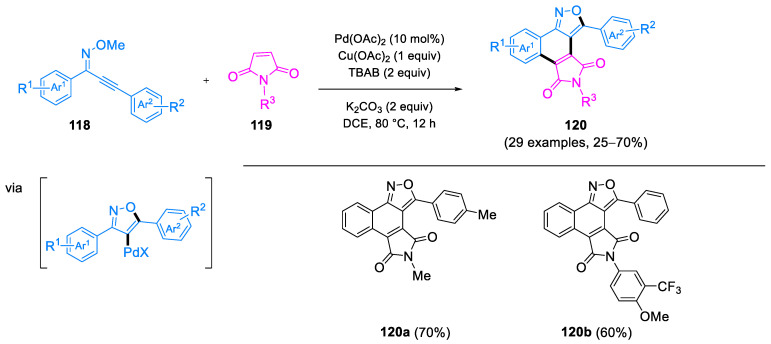
Pd-catalyzed synthesis of isoxazole–phthalimide-fused poly-heterocycles **120** via sequential cyclization and C–H anellation [88].

**Scheme 47:**
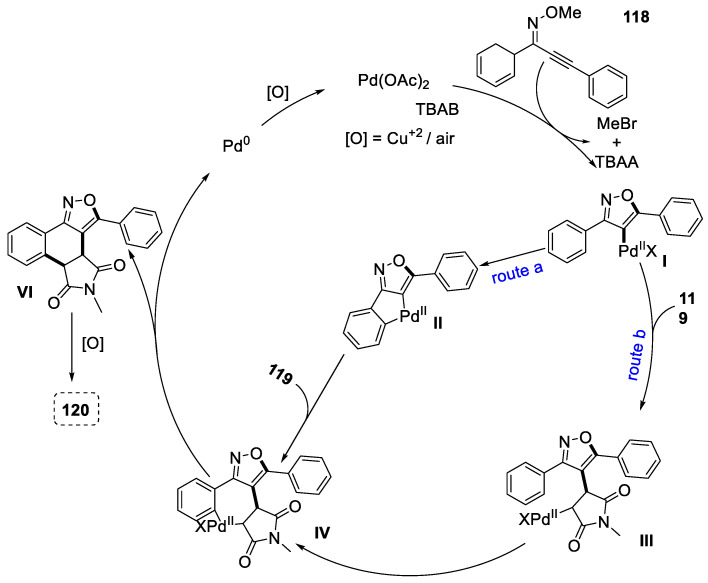
Proposed mechanism for palladium-catalyzed sequential electrophilic cyclization and C–H annellation cascade forming isoxazole–phthalimide-fused poly-heterocycles **120** [88].

Suresh and colleagues demonstrated a highly regioselective approach for synthesizing isoxazole–phthalimide-fused poly-heterocycles **120** through a sequential electrophilic cyclization and C–H arylation cascade [88]. While this is not a true sequence, it is another specially selected example meant to demonstrate the capabilities of the methodology in general: an advanced approach where directed C–H arylation finalizes the sequence, yielding a highly annulated aromatic system. The cascade, forming three bonds and two rings, efficiently utilizes organopalladium intermediates, which are crucial in facilitating the formation of C–C bonds between alkynyl-oxime ethers **118** and maleimides **119**, while copper species seemingly play an important assisting role (Scheme 46). The study also provides detailed mechanistic insights, including the exploration of kinetic isotope effects (KIE) and labeling experiments, revealing the stepwise nature of palladium migration and insertion processes (Scheme 47).

### 2.6. Sequentially Pd-Catalyzed Processes Based upon Miscellaneous Reactions

This section discusses cases that are not unambiguously categorized according to the major Pd-catalyzed processes but include key steps where Pd-catalyzed reactions and sequences play an essential role. Among these sequences are also those that particularly rely on reduction, oxidation, or amination.

**Scheme 48:**
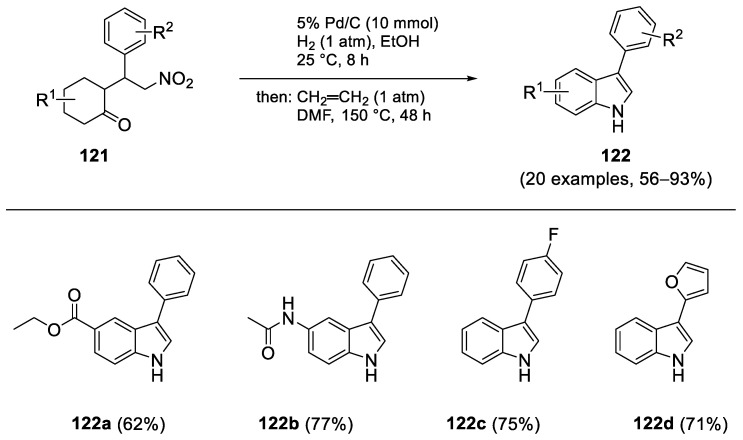
One-pot Pd/C-catalyzed synthesis of 3-substituted indoles **122** from 2-(2-nitro-1-phenylethyl)cyclohexanone derivatives **121** [89].

**Scheme 49:**
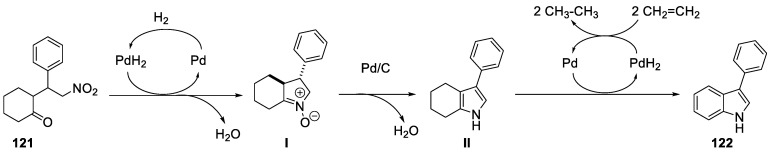
A tentative mechanism for the formation of indole **122** from 5-keto nitroalkanes **121** [89].

Kuang et al. developed a Pd/C-catalyzed one-pot method for synthesizing 3-substituted indoles **122** [89]. This approach employs a straightforward sequence of hydrogenation, cyclization, and dehydrogenation, starting from readily available 2-(2-nitro-1-phenylethyl)cyclohexanone derivatives **121** (Scheme 48 and Scheme 49). The method is particularly noteworthy for its high efficiency and chemoselectivity in indole formation, offering a streamlined route to these valuable heterocyclic compounds.

**Scheme 50:**
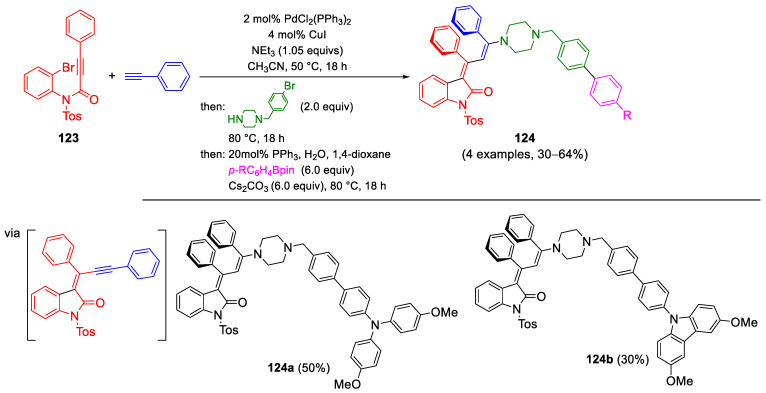
Consecutive four-component synthesis of 3-piperazinyl propenylidene indolone bichromophores **124** by Sonogashira alkynylation–alkyne insertion–hydroamination–Suzuki coupling sequence [90].

Merocyanine–triarylamine bichromophores **124** are synthesized via a sequential Pd-catalyzed Sonogashira alkynylation–alkyne insertion–hydroamination–Suzuki coupling four-component reaction sequence [90]. The resulting bichromophores exhibit white light emission upon aggregation in specific CH_2_Cl_2_–cyclohexane mixtures, attributed to aggregation-induced dual emission (AIDE) and partial energy transfer between chromophore units. The sequential Pd-catalyzed process facilitates the efficient production of bichromophores with yields ranging from 30 to 64% after a single purification step (Scheme 50). This synthetic protocol requires no additional catalyst loading after the initial catalytic step, ensuring high bond-forming efficiency. The bichromophores notably display dual aggregation-induced emission with blue and orange–red fluorescence, which is perceived as white light in specific solvent mixtures. 

**Scheme 51:**
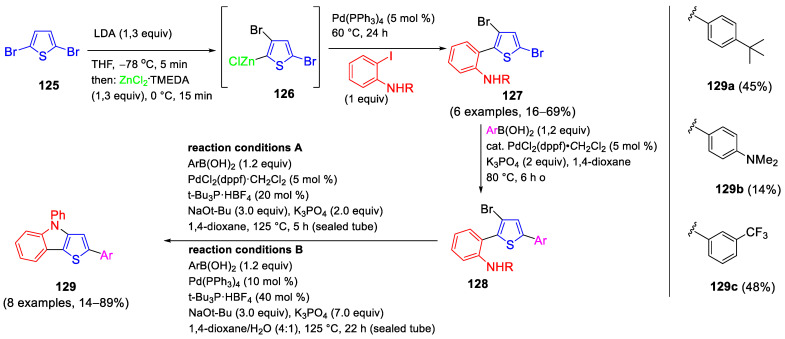
Synthesis of thieno[3,2-*b*]indoles **129** via “halogen dance”–Negishi–Suzuki–Buchwald–Hartwig sequence, with the couplings and amination performed under ligand-controlled conditions [91,92].

An exciting one-pot methodology for synthesizing anellated heterocycles, specifically aryl-substituted thieno[3,2-*b*]indoles **129**, is demonstrated via a Negishi–Suzuki-amination sequence that incorporates a “halogen dance” (i.e., a halogen shift) [91,92]. This unique feature makes the sequence noteworthy, as the halogen dance is used as a means to install the configuration of reactive sites for assembling the pyrrole moiety. In a preliminary step, the organozinc precursor is prepared from 2,5-dibromothiophene **125** by the action of LDA at −78 °C, which promotes a halogen shift (Scheme 51). After the addition of ZnCl_2_·TMEDA, 3,5-dibromothiophen-2-ylzinc chloride **126** is obtained, creating a third functionalizable position. Negishi **127** and ligand-controlled Suzuki couplings **128** are followed by a final intramolecular Buchwald–Hartwig amination to construct the central N-substituted pyrrolic ring **129**.

The key to efficient, regioselective conversion lies in in situ catalyst generation via controlled sequential ligand exchange, ensuring the selective formation of the desired C-C and C-N bonds and enabling the formation of targeted *π*-conjugated systems. The meticulous choice of ligands, particularly the synergistic combination of DPPF (1,1′-bis(diphenylphosphano)ferrocene, a bidentate ligand) and *t*-Bu_3_P (tri-*tert*-butylphosphane, a monodentate ligand), is crucial for controlling reactivity and selectivity during the synthesis. This approach is highly adaptable for creating various functionalized heterocyclic compounds with potential applications in material science and pharmaceuticals.

**Scheme 52:**
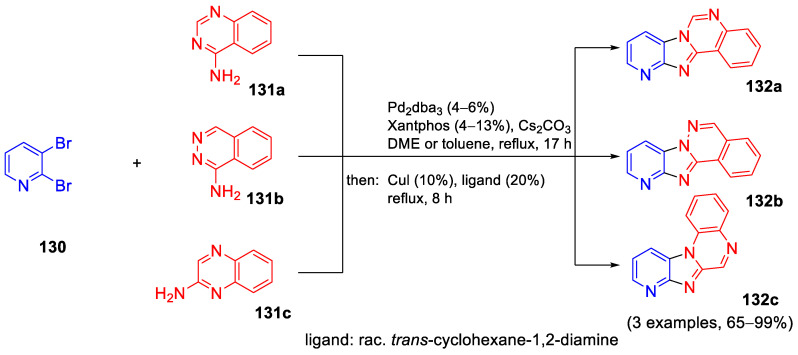
One-pot Pd/Cu-catalyzed sequential amination reaction for the synthesis of different tetraazabenzofluorenes **132** from 2,3-dibromopyridine **130** and benzodiazinamines **131** [93].

Rauws and coauthors introduced a sequential Pd/Cu-catalyzed one-pot methodology that relies on dual aminations for synthesizing complex tetracyclic azaheteroaromatic compounds **132** [93]. This process involves an initial Pd-catalyzed intermolecular amination of 2,3-dibromopyridine **130** with various benzodiazinamines **131**, followed by a Cu-catalyzed intramolecular cyclization, both essential for double C-N bond formation (the addition of copper seems to play an assisting function for the primary Pd catalyst) (Scheme 52). The team optimized the conditions by employing Pd_2_(dba)_3_ and Xantphos for the palladium-catalyzed step and CuI in conjunction with *rac-trans*-cyclohexane-1,2-diamine as a ligand for the copper-catalyzed cyclization. This sequential catalytic process proceeds under relatively mild conditions, achieving high yields (65–99% for three examples) and significant regioselectivity.

## 3. Sequentially Co-Catalyzed Processes with Palladium

In this final section, we explore recent advancements in sequential one-pot syntheses that leverage the synergistic effects of palladium and other metals (Pd,M catalysis). These methodologies underscore the potential of combining different catalytic systems to construct complex molecular architectures efficiently and with high selectivity. By integrating multiple catalytic steps within a single reaction vessel, these approaches not only streamline synthetic processes but also open new avenues for the synthesis of biologically relevant and structurally diverse heterocycles. The studies discussed below exemplify how dual-metal catalysis can enhance reaction efficiency, expand substrate scope, and provide access to novel compounds with significant potential in various fields, including medicinal chemistry and materials science.

**Scheme 53:**
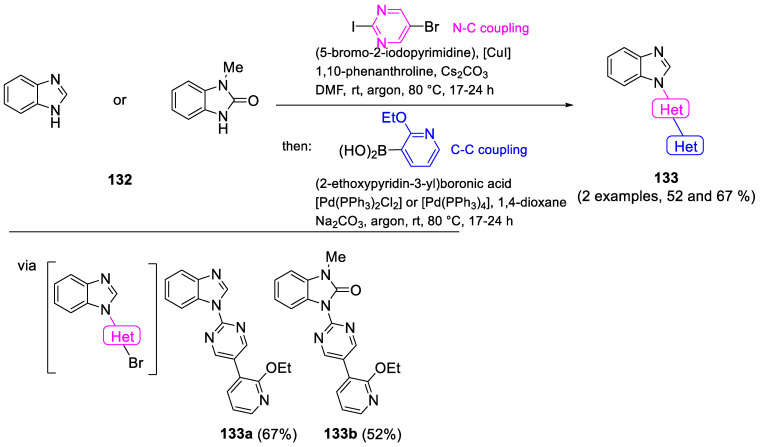
Construction of multi-heteroaryl scaffolds **133** via sequential N–C heteroarylation and Suzuki–Miyaura C–C cross-coupling reactions with aryl/heteroaryl boronic acids [94].

Siddle, Batsanov, and Bryce present a sequential one-pot synthesis that integrates copper-catalyzed N-heteroarylation with palladium-catalyzed C–C cross-coupling reactions [94]. This methodology is designed to construct tris(hetero)aryl systems **133** featuring two or three N-heterocyclic rings (Scheme 53). The process starts with copper-catalyzed N-arylation of benzimidazole **132**, imidazole, and pyrrole derivatives with heteroaryl iodides under modified Ullmann conditions. This step selectively forms N–C bonds, using Cs_2_CO_3_ as the base and CuI/1,10-phenanthroline as the catalyst system. The resulting N-heteroarylated intermediates are subsequently subjected to Suzuki–Miyaura cross-coupling reactions with aryl or arylboronic acids to form the final C–C bonds, completing the tris(hetero)aryl scaffold. The feasibility of conducting these reactions in a single-pot setup was demonstrated for two examples, as shown in Scheme 53. This one-pot process demonstrates a streamlined approach to synthesizing complex heteroaryl structures.

**Scheme 54:**
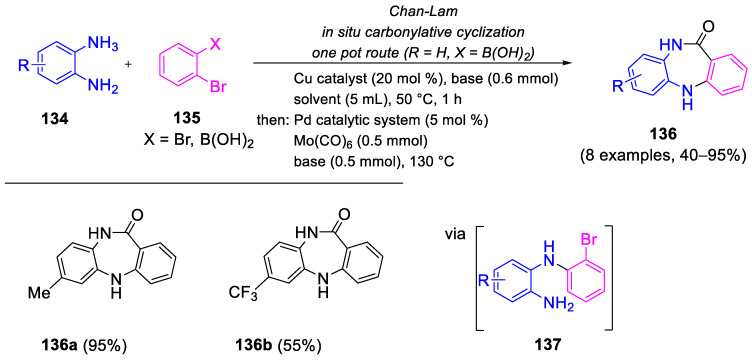
Sequential synthesis of 10,11-dihydro-5*H*-dibenzo[*b*,*e*][1,4]diazepinones **136** (DBDAPs) via copper-catalyzed Chan–Lam or Buchwald–Hartwig amination followed by palladium-catalyzed intramolecular aminocarbonylation using Mo(CO)_6_ as a CO surrogate [95].

**Scheme 55:**
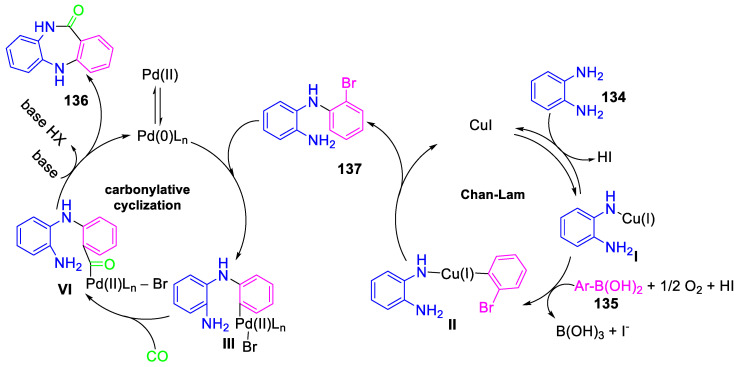
Proposed mechanism for Pd/Cu-catalyzed aminocarbonylation in the synthesis of dibenzodiazepinones [95].

Expanding on the versatility of Pd,M catalysis, Moutayakine and Burke presented a streamlined sequential synthesis of 10,11-dihydro-5*H*-dibenzo[*b*,*e*][1,4]diazepinones **136** (DBDAPs) using a Pd-catalyzed methodology [95]. The process begins with a Cu(II)-catalyzed Chan–Lam coupling of *o*-haloanilines **134** with aryl boronic acids **135**, followed by Pd-catalyzed Buchwald–Hartwig carbonylative cyclization, employing Mo(CO)_6_ as a CO surrogate (Scheme 54 and Scheme 55). This innovative approach enables the efficient formation of two C–N bonds and a C–C bond via the in situ generation of an *o*-(2-bromophenyl)aminoaniline intermediate **137**. Although the one-pot methodology successfully yielded the desired product in some instances, it was not effective for all substrates, necessitating a reliance on a stepwise approach for certain substrates. Nevertheless, this method represents a significant improvement over traditional multistep processes, providing a more efficient and streamlined synthesis of compounds such as dibenzepine derivatives, including the antidepressant clozapine.

**Scheme 56:**
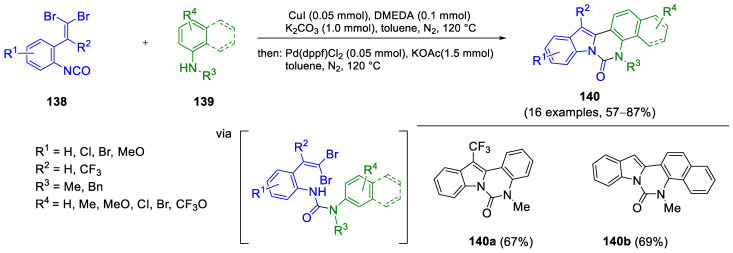
Sequential synthesis of pyrimido[1,6-*a*]indol-1(2*H*)-one derivative **140** via nucleophilic addition, Cu-catalyzed N-arylation, and Pd-catalyzed C-H activation [96].

Wang et al. presented a convenient one-pot protocol for synthesizing pyrimido[1,6-*a*]indol-1(2*H*)-one derivative **140** through a sequence of nucleophilic addition, Cu-catalyzed N-arylation, and Pd-catalyzed C-H activation [96]. The process used *ortho*-gem-dibromovinyl isocyanates **138** and *N*-alkyl-anilines **139** as substrates. After the initial nucleophilic addition **138**, a CuI-catalyzed N-arylation step was conducted using DMEDA as a ligand and K_2_CO_3_ as a base at 120 °C (Scheme 56). The reaction concluded with Pd(dppf)Cl_2_-catalyzed C-H activation facilitated by KOAc. This efficient method produced indole derivatives in moderate to good yields, demonstrating its utility for synthesizing biologically significant molecules.

Building on their previous work, Z.-J. Wang and colleagues describe a novel and efficient one-pot method for synthesizing unsymmetrical 2,2′-bi-indolyl derivatives via a sequential Cu-catalyzed N-arylation and Pd-catalyzed directed arylation process [97]. The key advancement in this study is the use of new starting materials, specifically *o*-gem-dibromovinylaniline and indole-1-carboxylic acid. Initially, indole-1-carboxylic acid was treated with oxalyl chloride to form the corresponding acid chloride, which then reacted with *o*-gem-dibromovinylaniline to afford the desired acylation product. The CuI-catalyzed N-arylation step was carried out under similar conditions as in their previous study, using DMEDA as a ligand and K_2_CO_3_ as a base in toluene at 120 °C. This was followed by a Pd(dppf)Cl_2_-catalyzed intramolecular directed arylation with KOAc as a base under the same conditions. This sequential process efficiently produced unsymmetrical 1,1′-carbonyl-2,2′-bi-indolyls in moderate-to-good yields, showcasing its applicability for synthesizing complex heterocyclic frameworks with potential biological activity.

**Scheme 57:**
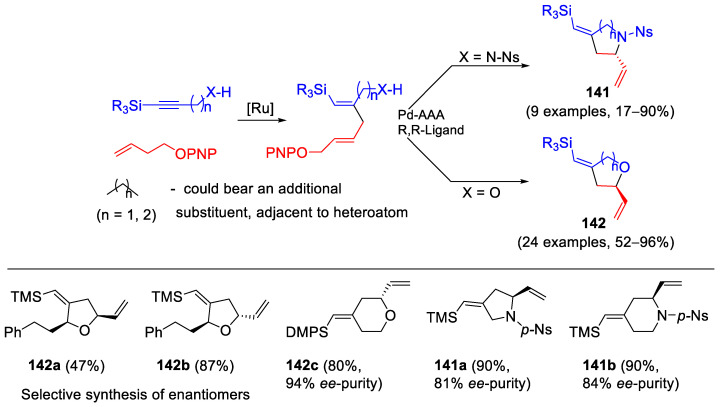
Sequential Ru-Pd catalysis: a one-pot dual-catalyst strategy for the synthesis of N- **141** and O-heterocycles **142** [98].

The work by Trost and co-authors introduces a sequential one-pot Ru-Pd catalyzed synthesis for constructing N- **141** and O-heterocycles **142** [98]. The method integrates a Ru-catalyzed alkene–alkyne Alder–ene addition with a subsequent Pd-catalyzed cyclization, producing enantio- and diastereopure heterocyclic compounds under mild conditions in a single reaction vessel (Scheme 57). The process is notable for its regio- and stereoselectivity, facilitating the synthesis of both *cis*- and *trans*-configured products, which are typically challenging to obtain through cyclization routes due to thermodynamics constraints. Their study also highlights the importance of the nucleophile in determining the stereochemistry of the asymmetric allylic cyclization reaction.

For “soft” nucleophiles like sulfonamides, the stereochemistry is controlled by kinetic trapping of the initially generated π–allyl complex. In contrast, for “hard” nucleophiles such as non-stabilized alcohols, selectivity is governed by slow trapping of equilibrating π–allyl diastereomers. This methodology’s utility was further illustrated through the concise synthesis of biologically relevant molecules, including intermediates for the total synthesis of kainoids and other natural products.

**Scheme 58:**
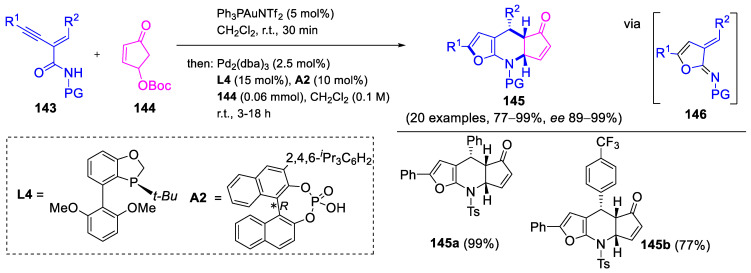
Sequential gold, palladium–phosphoric acid catalysis for the asymmetric synthesis of enantioenriched furo[2,3-*b*]pyridines **145** [99] (* indicates axial chirality).

**Scheme 59:**
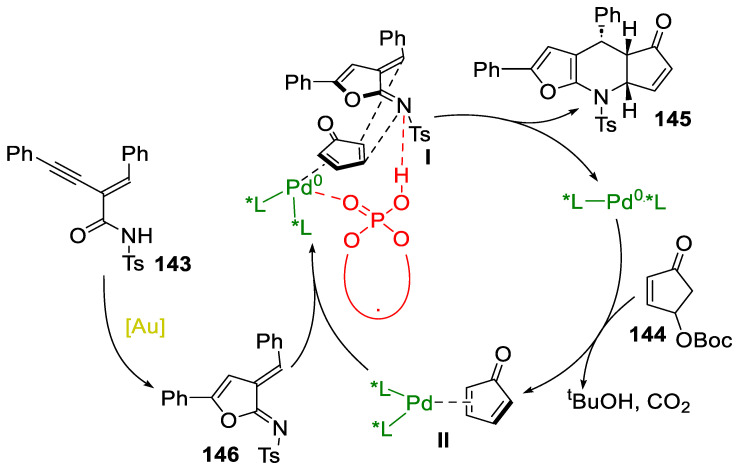
Proposed mechanism for sequential gold–palladium-catalyzed [4 + 2] cycloaddition in the synthesis of furo[2,3-*b*]pyridines **145** [99] (L* indicates chiral ligand **L4**).

Another exemplary case of tandem dual catalysis involving gold and palladium is demonstrated by Xie et al., who employ these metals in combination with chiral phosphoric acid to achieve a highly enantioselective [4 + 2] cycloaddition [99]. The process begins with gold-catalyzed cycloisomerization of ynamides **143** to generate reactive 1-azadiene intermediates **146** (Scheme 58). These intermediates then undergo a palladium-catalyzed cycloaddition with 4-hydroxy-2-cyclopentenone carbonate, during which an in situ formed HOMO-raised η^2^-Pd(0)–cyclopentadienone complex interacts with the azadiene species, leading to the formation of enantioenriched furo[2,3-*b*]pyridine derivatives **145**. This method exemplifies the power of multi-catalysis in constructing complex molecular architectures with high stereocontrol. The research team has previously published multiple studies that shed light on the mechanisms underlying these processes, making a significant contribution to the field of tandem catalysis (Scheme 59) [100,101].

**Scheme 60:**
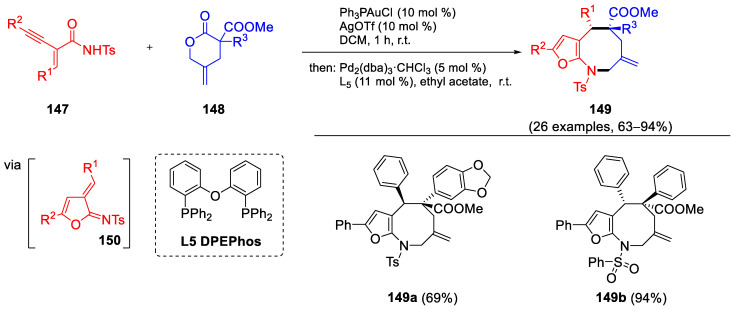
Efficient and diastereoselective gold–palladium relay catalysis system for the synthesis of fused eight-membered heterocycles **149**, starting with enynamides **147** that cyclize to form azadienes in situ, followed by [4 + 4] cycloadditions with γ-methylene-δ-valerolactones **148** [102].

**Scheme 61:**
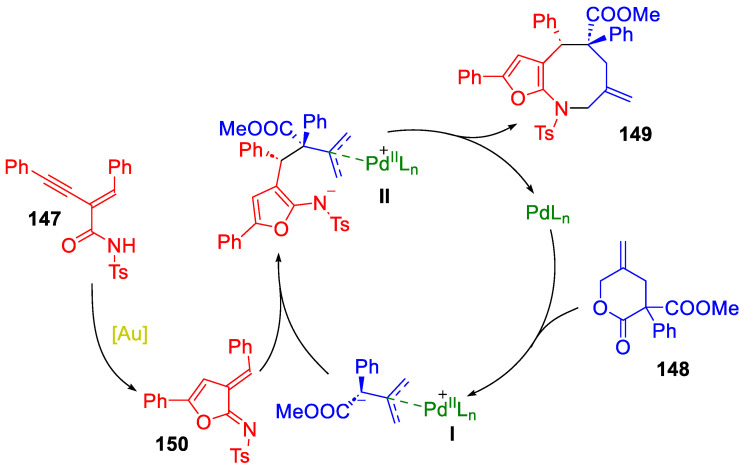
Proposed mechanism for gold–palladium relay catalytic [4 + 4] cycloadditions of enynamides and γ-methylene-δ-valerolactones [102].

Continuing this exploration, Gao et al. contribute to the growing field of palladium and gold relay catalysis, showing how this combination can be effectively used in a [4 + 4] cycloaddition to synthesize complex heterocycles **149**, further expanding the application of dual-metal systems in organic synthesis (Scheme 60 and Scheme 61) [102].

**Scheme 62:**
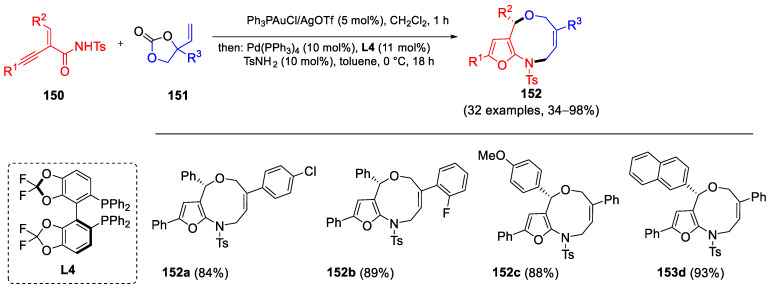
Stereoselective synthesis of polyfunctionalized nine-membered heterocycles **152** via sequential gold and palladium catalysis [103].

Yang, Ke, and Zhao report a stereoselective one-pot method for synthesizing polyfunctionalized furan-fused nine-membered heterocycles **152** using sequential gold and palladium catalysis [103]. The process begins with the gold-catalyzed cyclization of enynamides **150** to form azadienes in situ, which then undergo enantioselective formal [5 + 4] cycloaddition with vinyl ethylene carbonates **151** in the presence of a palladium catalyst (Scheme 62). This method demonstrates high efficiency and enantioselectivity, yielding densely functionalized nine-membered lactams and showcasing its potential for creating biologically significant medium-sized rings.

**Scheme 63:**
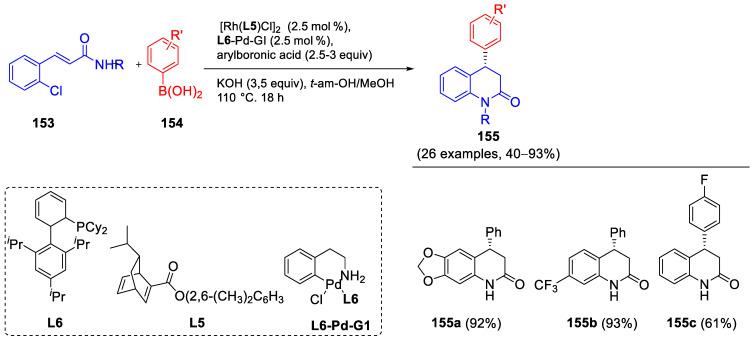
Sequential Rh/Pd catalysis with compatible achiral and chiral ligands enables highly enantioselective domino reactions, providing access to enantioenriched dihydroquinolinones **155** [104].

In this study, L. Zhang and M. Lautens introduce a versatile “two-catalysts-two-ligands” strategy for synthesizing chiral dihydroquinolinones **155** [104]. The reaction sequence begins with a Rh(I)-catalyzed enantioselective conjugate addition of acrylamides **153** to phenylboronic acid **154**, utilizing a chiral Hayashi diene ligand for high enantioselectivity (Scheme 63). This step is followed by a Pd-catalyzed C-N coupling via Buchwald–Hartwig amidation, facilitated by the L6-Pd-G1 catalyst with the biaryl phosphine ligand XPhos. This method efficiently produces enantioenriched dihydroquinolinones with high yields (up to 89%) and excellent enantioselectivities (up to 95%).

**Scheme 64:**
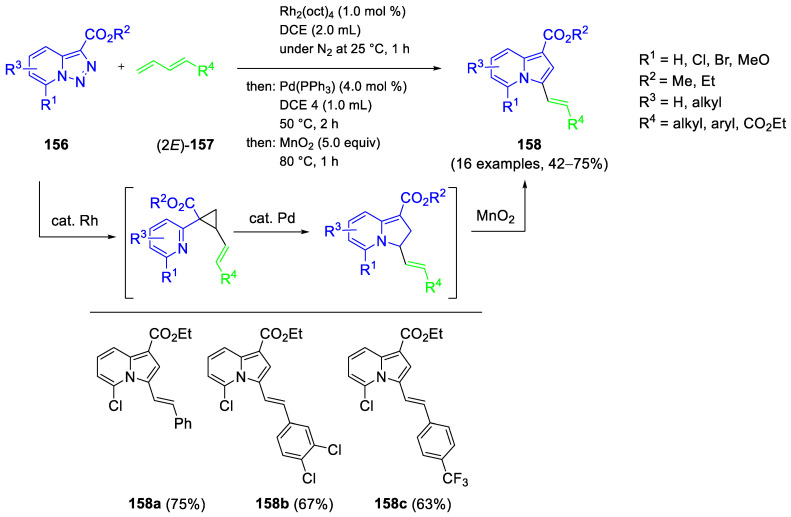
Synthesis of indolizine derivatives **158** via metal-catalyzed trans-annellations of pyridotriazoles **156** [105].

Kim et al. reported a novel sequential one-pot synthesis of indolizines **158**. This approach involves a Rh-catalyzed [2 + 1]-cyclopropanation of pyridotriazoles **156** with 1,3-dienes **157**, followed by a Pd-catalyzed ring expansion and oxidation [105]. The cyclopropanation was carried out with Rh_2_(oct)_4_ in DCE (1,2-dichloroethane), generating cyclopropane intermediates under mild conditions (25 °C). These intermediates were then converted into dihydroindolizines via Pd-catalyzed ring expansion using Pd(PPh_3_)_4_ at 50 °C and finally oxidized to indolizines using MnO_2_ at 80 °C (Scheme 64). This method exhibits excellent substrate compatibility, accommodating a range of electron-donating and electron-withdrawing groups on the aryl rings of the 1,3-dienes, yielding up to 75% of the final indolizine products.

**Scheme 65:**
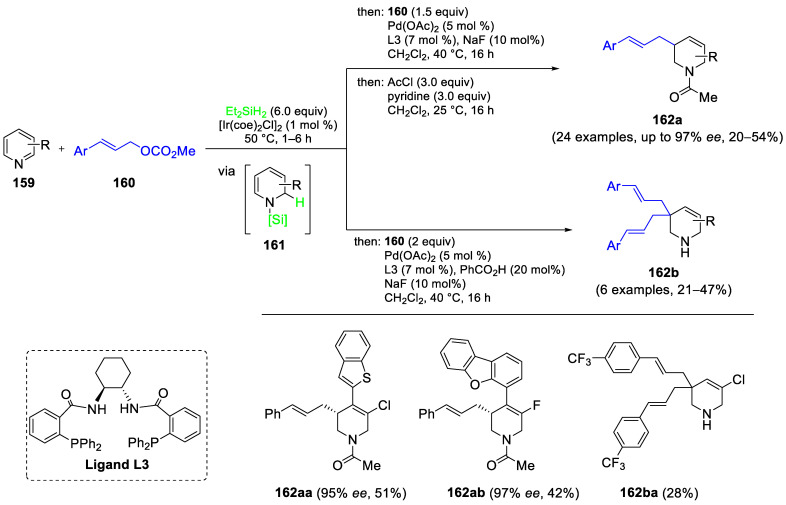
Iridium- and palladium-catalyzed tandem dearomatization and enantioselective allylic alkylation of pyridines **159** [106].

**Scheme 66:**
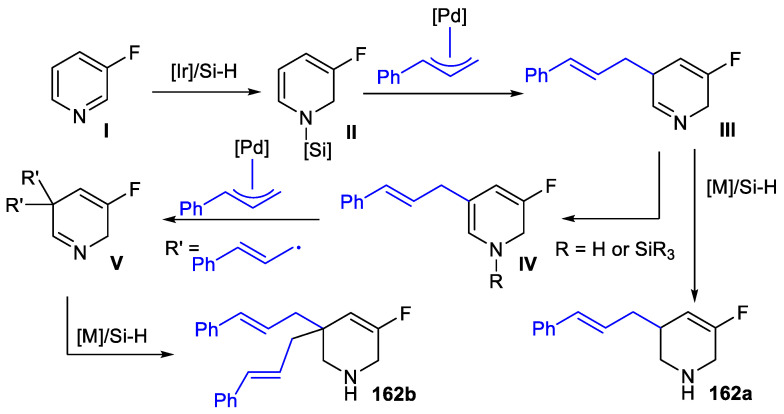
Proposed mechanism for iridium–palladium-catalyzed tandem dearomatization and asymmetric allylic alkylation [106].

Greßies et al. report a multistep, one-pot reaction for synthesizing N-protected tetrahydropyridines **162** with remarkable enantioselectivity (up to 97% *ee*) [106]. The method combines iridium(I)-catalyzed dearomative 1,2-hydrosilylation of pyridines **159** with a palladium-catalyzed asymmetric allylic alkylation **160** (Scheme 65 and Scheme 66). This approach employs N-silyl enamines **161** as a novel type of nucleophile, effectively overcoming the inherent nucleophilic selectivity of pyridines to access enantioenriched, C-3-substituted tetrahydropyridines, compounds that have historically been challenging to synthesize.

**Scheme 67:**
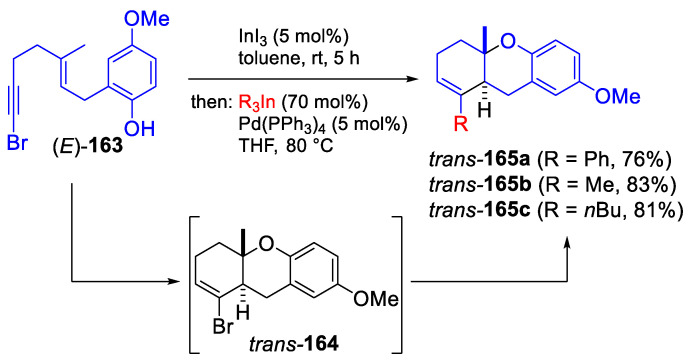
Sequential one-pot In-catalyzed 1,5-enyne cycloisomerization of (E)-**163** and subsequent Pd-catalyzed cross-coupling with triorganyl indium substrates [107].

The combination of indium(III) and palladium catalysis has proven highly effective for the sequential synthesis of tricyclic heterocycles **165** [107]. In the work of Millan et al., this catalytic duo drives a one-pot cascade of reactions, starting with an indium(III)-catalyzed stereoselective cycloisomerization of 1,5-enynes **163** followed by a palladium-catalyzed cross-coupling reaction (Scheme 67). The dual catalytic activity of In(III), acting as both σ-acid and π-acid facilitates efficient electrophilic activation of alkynes, leading to the selective *6-endo-dig* cyclization **164** and subsequent C–C hydroarylation or C–O phenoxycyclization, forming complex polycyclic frameworks such as benzo[*b*]chromenes, phenanthridines, xanthenes, and spiroheterocycles under mild conditions.

The double cyclization is stereospecific and operates via a biomimetic cascade cation-olefin mechanism. DFT studies support the formation of a nonclassical carbocation intermediate that evolves into the tricyclic product through a S_E_Ar mechanism. These insights illustrate the synergistic potential of indium(III) (cyclization) and palladium (triorganyl indium cross-coupling) catalysis in synthesizing advanced molecular architectures, with InI_3_ surpassing other indium halides in terms of yield and selectivity.

**Scheme 68:**
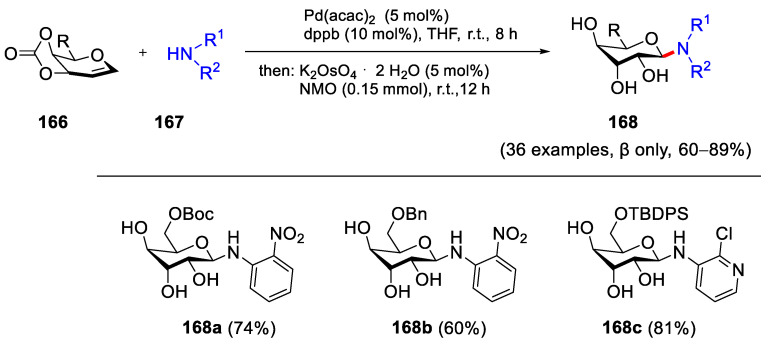
Synthesis of β-N-glycopyranosides **168** using a one-pot palladium-catalyzed Tsuji–Trost amination and dihydroxylation sequence at room temperature [108].

**Scheme 69:**
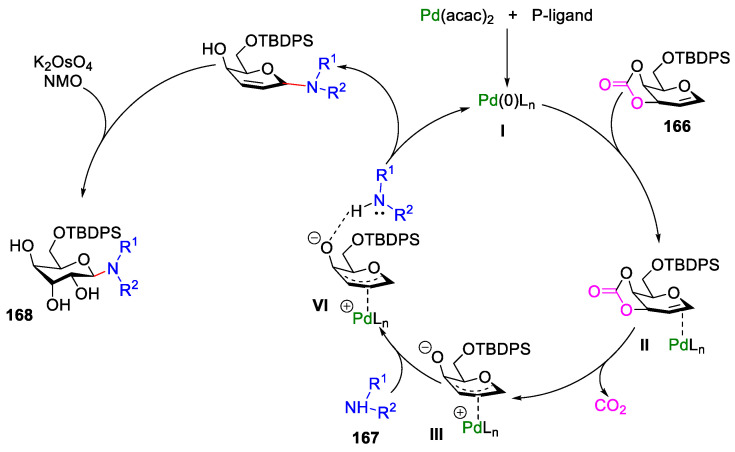
Proposed mechanism for palladium-catalyzed Tsuji–Trost amination followed by Sharpless dihydroxylation for stereoselective *N*-glycopyranoside synthesis [108].

Gao and colleagues reported a one-pot synthesis of 2,3,4-unprotected *β-N*-glycopyranosides **168** from glycals **166** and amines **167** with exclusive *β*-stereoselectivity at room temperature [108]. This method leverages palladium-catalyzed Tsuji–Trost amination, followed by direct dihydroxylation, and accommodating a wide range of substrates, including anilines, heterocyclic aromatic amines, and *N*,*O*-dimethylhydroxylamine (Scheme 68 and Scheme 69). The approach is particularly notable for its ability to facilitate reactions with primary amines and glycals, a combination that was previously unreported. The method has been successfully applied to the late-stage modification of clinical drugs like prazosin and imiquimod, as well as for synthesizing an analog of the natural product amphimedoside A.

**Scheme 70:**
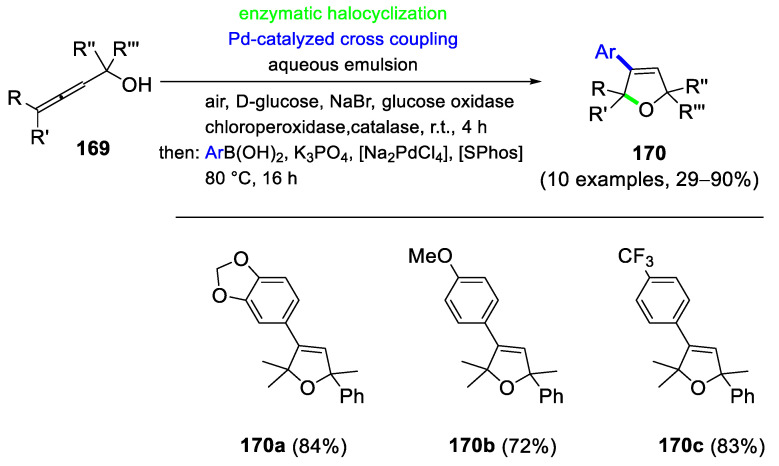
Arylative allenol cyclization via sequential one-pot enzyme and palladium catalysis [109,110].

The research conducted by Naapuri and colleagues introduces a sequential one-pot method integrating enzyme-mediated halogenation with palladium-catalyzed Suzuki-type cross-coupling [109,110]. This technique enables the direct arylative cyclization of allenic alcohols **169** with boronic acids, efficiently synthesizing unsaturated five-membered O-heterocycles **170**. The process begins with a biocatalytic halocyclization, mediated by chloroperoxidase from *Cercospora fumago* and glucose oxidase from *Aspergillus niger*. This is followed by a palladium-catalyzed Suzuki-type cross-coupling within an aqueous emulsion. The enzymatic oxidative activation of simple halide salts serves as a traceless ring-closure-inducing event, triggering the subsequent C−C coupling. Operating under mild conditions with air as the terminal oxidant, this approach produces densely substituted dihydrofurans in high yields (Scheme 70). The inclusion of a nanobiohybrid catalyst, consisting of palladium nanoparticles embedded in a glucose oxidase matrix, enhances overall catalytic performance, allowing for the effective synthesis of highly functionalized O-heterocycles. This enzyme–metal hybrid not only supports biocyclization but also promotes the C−C bond formation via cross-coupling, showcasing the potential of this innovative technique for streamlining chemoenzymatic methodologies. The robustness of biocatalytic halogenations is exemplified by successful combinations with both Suzuki and Sonogashira couplings. 

## 4. Conclusions

Sequentially Pd-catalyzed one-pot syntheses represent a transformative approach in the field of organic chemistry, offering efficient, selective, and sustainable routes to complex heterocyclic derivatives. These methodologies offer significant advantages, including enhanced atom economy, reduced reaction times, and minimized waste, making them highly attractive for both academic research and industrial applications. The integration of palladium catalysis with other metals, such as copper, ruthenium, and gold, has further expanded the scope and versatility of these processes, enabling the efficient construction of diverse and functionalized heterocyclic frameworks.

Despite the numerous benefits, challenges remain, including substrate limitations and the necessity for careful optimization of reaction conditions to achieve high yields and selectivity. However, the continuous evolution of these methods, particularly with the incorporation of novel catalysts and dual-metal systems, holds great promise for overcoming these challenges. Future research is expected to focus on expanding substrate scope, exploring new catalytic systems, and further enhancing the efficiency and sustainability of these reactions.

In conclusion, sequentially Pd-catalyzed one-pot methodologies are poised to play an increasingly important role in the synthesis of heterocyclic derivatives, especially in the development of pharmaceuticals and advanced materials. As these methods continue to evolve, they will undoubtedly contribute to green chemistry principles and sustainable synthesis, offering powerful tools for the efficient construction of complex molecular architectures.

## Data Availability

Not applicable.
