# Correction: Kornet, M.M.; Müller, T.J.J. Recent Advances in Sequentially Pd-Catalyzed One-Pot Syntheses of Heterocycles. *Molecules* 2024, *29*, 5265

**DOI:** 10.3390/molecules30091862

**Published:** 2025-04-22

**Authors:** Maryna M. Kornet, Thomas J. J. Müller

**Affiliations:** 1 Institut für Organische Chemie und Makromolekulare Chemie, Mathematisch-Naturwissenschaftliche Fakultät, Heinrich-Heine-Universität Düsseldorf, Universitätsstrasse 1, D-40225 Düsseldorf, Germany; 2 Laboratory for Biotechnology of Physiologically Active Substances, Faculty of Biology, Zaporizhzhia National University, 66 Universytetska Str., 69600 Zaporizhzhia, Ukraine

In the original publication [1], the funding statement is incomplete, the correct funding appears below: This project has received funding through the MSCA4Ukraine project, which is funded by the European Union. The views and opinions expressed are those of the author(s) only and do not necessarily reflect those of the European Union. Neither the European Union nor the MSCA4Ukraine Consortium as a whole nor any individual member institutions of the MSCA4Ukraine Consortium can be held responsible for them. In addition, support by Fond der Chemischen Industrie (ad personam support of T.J.J.M.) is acknowledged.

The authors state that the scientific conclusions are unaffected. This correction was approved by the Academic Editor. The original publication has also been updated.
